# C-reactive protein–triglyceride–glucose index and diabetes status for risk stratification in stage 4 cardiovascular–kidney–metabolic syndrome with acute decompensated heart failure: a retrospective cohort study

**DOI:** 10.3389/fnut.2026.1903670

**Published:** 2026-09-09

**Authors:** Wei Wang, Jiaming Zhang, Miaoxin Chen, Yu Wang, Xingyi Lai, Rong Huang, Lei Hu, Lian Wang

**Affiliations:** 1 Department of Cardiology, Nanjing Drum Tower Hospital, Affiliated Hospital of Medical School, Nanjing University, Nanjing, Jiangsu, China; 2 Department of Cardiology, Nanjing Drum Tower Hospital, Nanjing University of Chinese Medicine, Nanjing, Jiangsu, China; 3 Department of Cardiology, Shanghai Institute of Cardiovascular Diseases, Zhongshan Hospital, Fudan University, Shanghai, China

**Keywords:** acute decompensated heart failure, cardiovascular–kidney–metabolic syndrome, C-reactive protein–triglyceride–glucose index, diabetes status, metabolic inflammation, risk stratification

## Abstract

**Background:**

Cardiovascular–kidney–metabolic (CKM) syndrome integrates metabolic dysfunction, kidney disease, and cardiovascular disease. In stage 4 CKM syndrome with acute decompensated heart failure (ADHF), prognosis may be influenced by dysglycaemia, triglyceride-related metabolic disturbance, systemic inflammation, and adiposity-related risk, yet risk stratification remains challenging. We evaluated whether the C-reactive protein–triglyceride–glucose index (CTI), a routinely available inflammatory-metabolic marker, refines risk stratification beyond diabetes status and related inflammatory-metabolic biomarkers in patients with stage 4 CKM syndrome and ADHF.

**Methods:**

This single-centre retrospective cohort included 2,000 eligible patients hospitalized for ADHF with CKM stage 4. CTI was calculated as 0.412 × ln[CRP (mg/L)] + ln[TG (mg/dL) × FPG (mg/dL) / 2]. High CTI was defined using the cohort-specific upper-tertile threshold (CTI ≥ 9.50). Patients were categorized as non-DM + low CTI, non-DM + high CTI, DM + low CTI, or DM + high CTI. Outcomes were all-cause mortality and major adverse cardiac and cerebrovascular events (MACCEs).

**Results:**

During a median follow-up of 532 days, 366 deaths (18.3%) and 551 MACCEs (27.6%) occurred. Compared with non-DM + low CTI, non-DM + high CTI was associated with higher risks of all-cause mortality (HR 1.54, 95% CI 1.15–2.06) and MACCEs (HR 1.37, 95% CI 1.08–1.75). DM + low CTI was not significantly associated with either outcome, whereas DM + high CTI had the highest risks of all-cause mortality (HR 1.95, 95% CI 1.47–2.58) and MACCEs (HR 1.91, 95% CI 1.51–2.41). CTI showed approximately linear associations with both outcomes, without significant interaction by diabetes status. Adding CTI to the clinical model plus diabetes status yielded modest improvements in discrimination (ΔC-index = 0.018 for mortality and 0.016 for MACCEs), exceeding those of the evaluated biomarker models. Sensitivity analyses generally supported the primary findings.

**Conclusion:**

In patients with stage 4 CKM syndrome hospitalized for ADHF, elevated CTI was associated with higher risks of all-cause mortality and MACCEs, with the greatest risk in patients with both diabetes and high CTI. CTI may be a candidate adjunctive marker for risk refinement; external validation is required before clinical implementation.

## Graphical abstract

**Figure:**
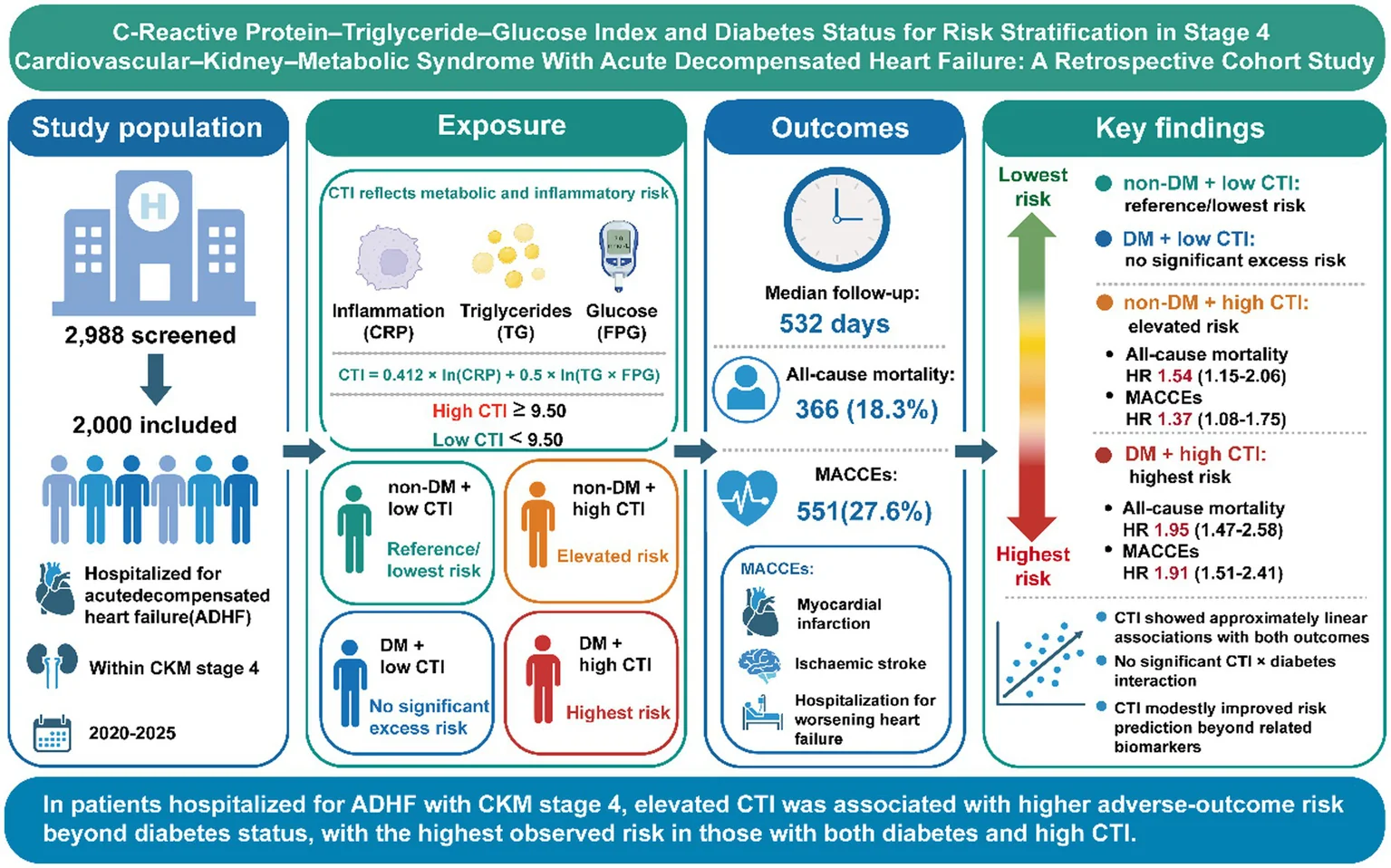
In patients hospitalized for ADHF with CKM stage 4, elevated CTI was associated with higher risk beyond diabetes status and related biomarkers, with highest risk in diabetes plus high CTI. Created in BioRender. Shuhui, W. (2026) https://BioRender.com/i6asewo.

## Introduction

Cardiovascular–kidney–metabolic (CKM) syndrome was recently proposed by the American Heart Association as an integrated framework describing the pathophysiological interplay among metabolic dysfunction, chronic kidney disease, and cardiovascular disease (1). Within this framework, CKM stage 4 represents an advanced clinical state characterized by established cardiovascular disease in the context of underlying cardiometabolic dysfunction and a high risk of adverse outcomes (1, 2).

Heart failure is a major clinical manifestation of CKM syndrome, and hospitalization for acute decompensated heart failure (ADHF) marks a particularly vulnerable period with high rates of early readmission and post-discharge mortality (2–4). Contemporary heart failure guidelines and recent reviews consistently emphasize the need for improved post-discharge risk stratification in this population (3, 4).

Because many patients hospitalized for ADHF have coexisting metabolic, renal, or adiposity-related abnormalities, operational CKM stage 4 may overlap substantially with contemporary real-world ADHF populations. In the present study, the CKM framework was therefore not used to define a rare or mutually exclusive heart failure subgroup; rather, it provided a structured cardiometabolic context in which residual prognostic heterogeneity after ADHF hospitalization could be examined.

Diabetes mellitus is a core component of the CKM construct and an established determinant of adverse heart failure outcomes (1, 5, 6). However, the presence of diabetes alone may not fully explain prognostic heterogeneity in patients hospitalized for ADHF. Even in advanced CKM stage 4, outcomes are likely shaped not only by dysglycaemia, but also by the broader inflammatory-metabolic milieu that accompanies insulin resistance, adiposity, endothelial dysfunction, renal impairment, and systemic inflammation (1, 7–9).

Accordingly, markers integrating glycaemic, lipid-related, and inflammatory information may refine risk assessment beyond diabetes status in advanced CKM syndrome.

Inflammation and insulin resistance are increasingly recognized as closely interrelated processes in the progression of cardiometabolic disease (7–9). The C-reactive protein–triglyceride–glucose index (CTI), first proposed as a composite biomarker integrating C-reactive protein with the triglyceride–glucose index, combines routinely available information on systemic inflammation, triglyceride metabolism, and fasting glycaemia (10). Since then, CTI has been associated with adverse cardiovascular and mortality outcomes across several populations (11–13). Within the CKM framework, integrated metabolic markers such as the triglyceride–glucose–body mass index (TyG-BMI), the atherogenic index of plasma (AIP), and CTI have also been associated with incident cardiovascular disease, stroke, and mortality in individuals with CKM stages 0–3 (11–16). These observations suggest that composite markers integrating inflammatory and metabolic information may be relevant to cardiovascular risk assessment within the CKM framework. However, whether these composite indices provide prognostic information beyond their individual components, particularly in patients hospitalized for ADHF within the advanced CKM spectrum, remains unclear.

We therefore examined the associations of diabetes status and CTI with all-cause mortality and MACCEs in patients hospitalized for ADHF with CKM stage 4 and compared the incremental prognostic value of CTI with that of CRP, TG, FPG, TyG, CRP + TyG, and CRP + TG + FPG.

## Methods

### Study population

**Figure 1:**
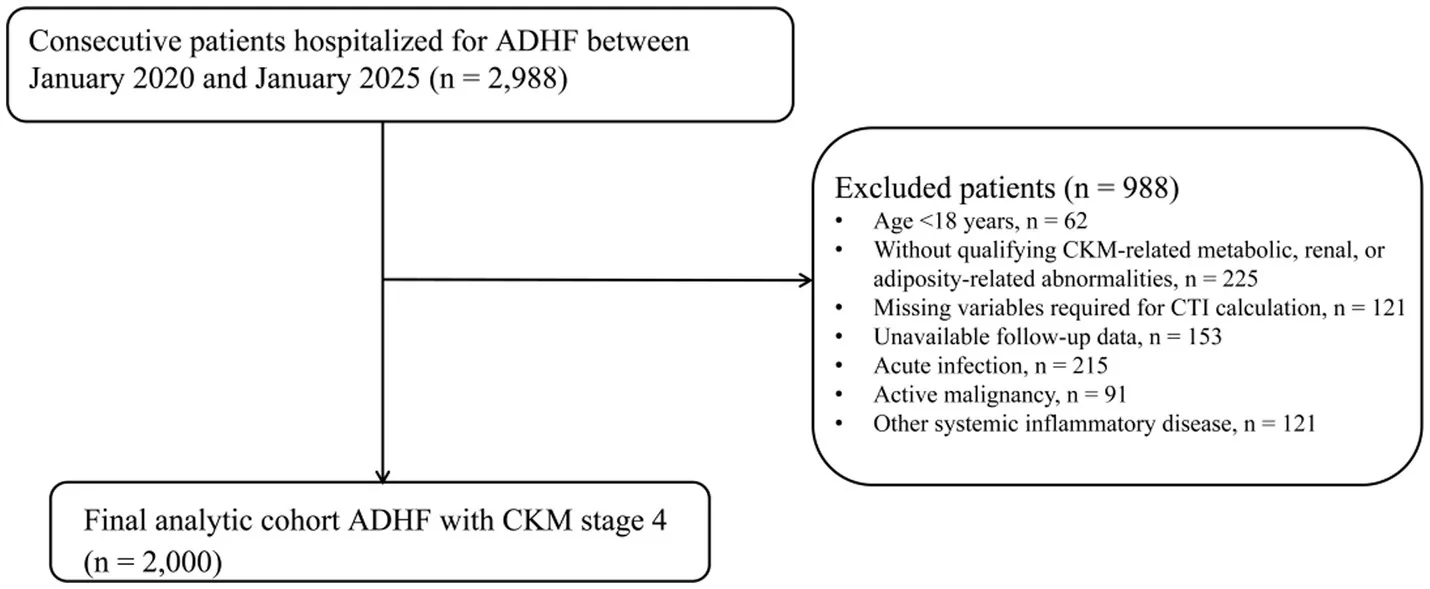
Flowchart of the study population. CKM stage 4 was operationally defined according to the AHA CKM staging framework as clinical cardiovascular disease, represented in this cohort by hospitalization for ADHF, plus at least one documented CKM-related metabolic, renal, or adiposity-related abnormality. Of 2,988 screened patients, 988 were excluded sequentially: Age <18 years (*n* = 62), no qualifying CKM-related abnormality (*n* = 225), missing CTI variables (*n* = 121), unavailable follow-up (*n* = 153), acute infection (*n* = 215), active malignancy (*n* = 91), or another systemic inflammatory disease (*n* = 121). ADHF, acute decompensated heart failure; CKM, cardiovascular–kidney–metabolic; CTI, C-reactive protein–triglyceride–glucose index; DM, diabetes mellitus.

This single-centre retrospective cohort study included consecutive patients hospitalized for acute decompensated heart failure (ADHF) at Nanjing Drum Tower Hospital, Affiliated Hospital of Medical School, Nanjing University, from January 2020 to January 2025. Patients were eligible if they fulfilled the criteria for cardiovascular–kidney–metabolic (CKM) syndrome stage 4 and had available data to calculate the C-reactive protein–triglyceride–glucose index (CTI). Of 2,988 screened patients, 988 were excluded sequentially because of age <18 years (*n* = 62), absence of a qualifying CKM-related metabolic, renal, or adiposity-related abnormality (*n* = 225), missing variables required for CTI calculation (*n* = 121), unavailable follow-up (*n* = 153), acute infection (*n* = 215), active malignancy (*n* = 91), or another systemic inflammatory disease (*n* = 121). The remaining 2,000 patients were included in the final analysis (Figure 1). The study was conducted in accordance with the Declaration of Helsinki and was approved by the institutional Ethics Committee.

### Definition of CKM stage 4 and CTI

CKM stage 4 was operationally defined according to the American Heart Association CKM staging framework. In this framework, stage 4 CKM syndrome refers to clinical cardiovascular disease in the setting of CKM syndrome; clinical cardiovascular disease includes heart failure, coronary heart disease, stroke, peripheral artery disease, or atrial fibrillation. Accordingly, hospitalization for acute decompensated heart failure was considered the established clinical cardiovascular disease component in the present retrospective inpatient cohort. To avoid classifying cardiovascular disease alone as CKM stage 4, patients were additionally required to have at least one documented CKM-related metabolic, renal, or adiposity-related abnormality, including diabetes or prediabetes, hypertension, hyperlipidaemia, chronic kidney disease, or excess/dysfunctional adiposity. Excess/dysfunctional adiposity was defined as BMI ≥ 25 kg/m^2^, consistent with the AHA CKM staging framework threshold for overweight/adiposity-related risk. Patients hospitalized for ADHF without any qualifying CKM-related abnormality were not classified as CKM stage 4 and were excluded from the analytic cohort.

CTI was calculated as 0.412 × ln[CRP (mg/L)] + ln[TG (mg/dL) × FPG (mg/dL) / 2]. The triglyceride–glucose index (TyG) was calculated as ln[TG (mg/dL) × FPG (mg/dL) / 2]. High CTI was defined as CTI ≥ 9.50, the cohort-specific upper-tertile threshold, and low CTI as CTI < 9.50. For the primary analysis, patients were classified into four groups according to clinically documented prior diagnosis of diabetes mellitus and CTI level: non-DM + low CTI, non-DM + high CTI, DM + low CTI, and DM + high CTI.

For descriptive supplementary analyses, CKM burden was calculated as the number of qualifying non-cardiovascular CKM abnormalities, including dysglycaemia, hypertension, hyperlipidaemia, chronic kidney disease, and adiposity abnormality as defined below. CKM burden was summarized as 1, 2, or ≥3 abnormalities.

### Outcomes and covariates

Two predefined primary endpoints were examined: (1) all-cause mortality, defined as death from any cause; and (2) major adverse cardiac and cerebrovascular events (MACCEs), defined as a non-fatal composite of myocardial infarction, ischaemic stroke, and hospitalization for worsening heart failure. Time-to-event for each outcome was calculated from the date of index hospitalization to the first occurrence of the outcome of interest. Patients who did not experience the outcome of interest were censored at the date of last confirmed contact recorded in the follow-up database or at the administrative end of follow-up, whichever occurred first.

Demographic, clinical, laboratory, echocardiographic, and medication data at admission were retrieved from the electronic medical record system by trained physicians. Body mass index (BMI) was calculated as weight in kilograms divided by height in meters squared. Excess or dysfunctional adiposity was operationally defined as BMI ≥ 25 kg/m^2^. Peripheral venous blood samples were collected after an overnight fast of at least 8 h and processed at the central laboratory according to standardized procedures. Laboratory measurements included haemoglobin, uric acid, fasting plasma glucose (FPG), glycated haemoglobin (HbA1c), total cholesterol (TC), triglycerides (TG), low-density lipoprotein cholesterol (LDL-C), high-density lipoprotein cholesterol (HDL-C), creatinine, estimated glomerular filtration rate (eGFR), C-reactive protein (CRP), B-type natriuretic peptide (BNP), white blood cell count (WBC), sodium, potassium, albumin (ALB), alanine aminotransferase (ALT), and aspartate aminotransferase (AST). Chronic kidney disease was defined as eGFR <60 mL/min/1.73 m^2^.

Hypertension was defined as systolic blood pressure (SBP) ≥ 140 mmHg, diastolic blood pressure (DBP) ≥ 90 mmHg, current use of antihypertensive medication, or a documented prior diagnosis. For the primary analysis, diabetes mellitus (DM) status was defined according to a clinically documented prior diagnosis; patients without a documented prior diagnosis of DM were classified as non-DM. For the operational definition of CKM stage 4 and the supplementary AHA glycaemic-category analysis, glycaemic status was further classified using FPG, HbA1c, and documented diabetes history: normal glucose regulation was defined as FPG < 100 mg/dL and HbA1c < 5.7% without documented diabetes; prediabetes was defined as FPG 100–125 mg/dL or HbA1c 5.7–6.4% without documented diabetes; and diabetes was defined as FPG ≥ 126 mg/dL, HbA1c ≥ 6.5%, or documented diabetes. Hyperlipidaemia was defined according to established guidelines as TC ≥ 240 mg/dL, LDL-C ≥ 160 mg/dL, TG ≥ 150 mg/dL, HDL-C < 40 mg/dL, or ongoing lipid-lowering therapy.

### Supplementary analysis using AHA glycaemic categories

In addition to the primary analysis based on prior diagnosis of DM, we performed a supplementary analysis using the American Heart Association glycaemic categories. Specifically, participants were classified as normoglycaemia, prediabetes, or diabetes according to fasting plasma glucose, HbA1c, and prior history of diabetes: normoglycaemia was defined as FPG < 100 mg/dL and HbA1c < 5.7% without a prior diagnosis of DM; prediabetes was defined as FPG 100–125 mg/dL or HbA1c 5.7–6.4% without a prior diagnosis of DM; and diabetes was defined as FPG ≥ 126 mg/dL, HbA1c ≥ 6.5%, or a prior diagnosis of DM. This analysis was designed as a complementary analysis rather than as a redefinition of the primary exposure.

### Statistical analysis

Continuous variables are presented as mean ± standard deviation or median (interquartile range), as appropriate, and categorical variables as number (percentage). Between-group differences were compared using one-way analysis of variance or the Kruskal–Wallis test for continuous variables and the *χ*^2^ test for categorical variables. Missing covariate data were handled using multiple imputation by chained equations under a missing-at-random assumption. B-type natriuretic peptide had the highest proportion of missing values (15.7%). Five imputed datasets were generated using predictive mean matching with 20 iterations. The imputation model included the exposure variables, Model 3 covariates, event indicators, and follow-up times, and regression coefficients and standard errors from the Cox models were pooled across the five imputed datasets according to Rubin’s rules.

CTI was categorized into tertiles according to its distribution in the overall cohort. High CTI was defined as CTI ≥ 9.50, whereas low CTI was defined as CTI < 9.50. Patients were classified into four groups according to diabetes status and CTI level: non-DM + low CTI, non-DM + high CTI, DM + low CTI, and DM + high CTI. The four groups were used to describe joint risk strata, whereas effect modification was assessed separately using formal interaction tests. Conventional Kaplan–Meier curves were generated to compare crude event-free survival across the four groups for all-cause mortality and MACCEs, and between-group differences were assessed using the log-rank test.

Cox proportional hazards models were used to estimate hazard ratios (HRs) and 95% confidence intervals (CIs) for the associations of the four diabetes/CTI groups with the two primary outcomes, using the non-DM + low CTI group as the reference group. Three hierarchical models were constructed: Model 1 was unadjusted; Model 2 was adjusted for age and sex; and Model 3 was further adjusted for BMI, SBP, HR, eGFR, LVEF, BNP, atrial fibrillation, coronary artery disease, and the use of SGLT2 inhibitors, ACEIs/ARBs/ARNIs, beta-blockers, MRAs, diuretics, and statins. Covariates were selected *a priori* based on clinical relevance and established associations with heart failure prognosis, and the models were intended for prognostic assessment rather than causal inference. Univariate associations and variance inflation factors were examined as supportive checks for model stability and collinearity. The proportional hazards assumption was assessed using Schoenfeld residuals.

CTI was also analysed as a continuous variable, and restricted cubic spline analyses were performed to assess dose–response relationships between CTI and study outcomes. Restricted cubic spline models were fitted with four knots placed at the 5th, 35th, 65th, and 95th percentiles of the CTI distribution and were adjusted for the same covariates as Model 3. The median CTI value was used as the reference. Tests for non-linearity were performed by comparing models with and without the non-linear spline terms. Formal interaction tests were performed to evaluate whether the association between CTI and outcomes differed according to diabetes status, with CTI modelled both as a continuous variable and as a high versus low categorical variable.

Exploratory subgroup analyses considered age, sex, body mass index, hypertension, left ventricular ejection fraction, and SGLT2 inhibitor use. Adjusted Cox models included multiplicative interaction terms. Benjamini–Hochberg false-discovery-rate (FDR) correction was applied to the 24 tests defined by six subgroup variables, two outcomes, and two exposure contrasts (DM + high CTI and non-DM + high CTI versus non-DM + low CTI); both nominal and adjusted *p* values were reported.

To evaluate whether CTI provided incremental prognostic information beyond diabetes status and related inflammatory and metabolic biomarkers, we compared a series of biomarker models using the clinical model plus diabetes status as the reference model. The evaluated models additionally incorporated CRP, TG, FPG, TyG, CRP plus TyG, CRP plus TG plus FPG, or CTI. A model incorporating the four groups defined by diabetes status and CTI level was also evaluated; this model included the same clinical covariates and the four-category diabetes/CTI grouping variable, without diabetes status entered separately. In a single completed imputed dataset comprising all 2,000 participants, model performance was assessed using Harrell’s C-index, the 1-year time-dependent area under the curve (AUC), likelihood-ratio tests, continuous net reclassification improvement (NRI), integrated discrimination improvement (IDI), Brier score, and calibration slope; calibration and decision-curve analyses were also performed at the prespecified 1-year horizon.

Sensitivity analyses comprised Fine–Gray competing-risk models, a 30-day landmark analysis, stratified Cox models, inflammatory-marker exclusion analyses, and a complete-case analysis. For the MACCE endpoint, death occurring before a MACCE was treated as a competing event. The landmark analysis was restricted to patients who were alive and free of the relevant outcome at 30 days and therefore estimated risk among 30-day event-free survivors. The primary models were repeated after separately excluding patients with CRP > 20 mg/L, CRP > 50 mg/L, or WBC > 10 × 10^9^/L, with the original CTI cut-off of 9.50 retained in each analysis. The complete-case analysis was restricted to participants with complete data for all Model 3 variables. All analyses were conducted using R version 4.5.2. A two-sided *p* < 0.05 was considered statistically significant.

## Results

### Baseline characteristics according to diabetes status and CTI level in CKM stage 4

Among the 2,000 patients, baseline characteristics differed across the four groups defined by diabetes status and CTI level. Overall, the non-DM + high CTI group exhibited a less favourable inflammatory-metabolic profile despite the absence of documented diabetes. By contrast, the DM + low CTI group showed dysglycaemia but without a similarly elevated inflammatory-metabolic profile (Table 1). To place these differences among groups in a broader glycaemic context, baseline characteristics were further examined according to the three AHA glycaemic categories. Across these categories, patients with diabetes showed a less favourable overall cardiometabolic and renal profile than those with normoglycaemia or prediabetes, supporting the higher baseline risk of this subgroup in the supplementary analyses (Supplementary Table S1).

**Table 1:** Baseline characteristics according to diabetes status and CTI level.

Variable	Overall	non-DM + low CTI	non-DM + high CTI	DM + low CTI	DM + high CTI	*p* value
*N*	2,000	907	332	417	344	
Male sex, *n* (%)	1,062 (53.1)	482 (53.1)	186 (56.0)	228 (54.7)	166 (48.3)	0.187
Age, years	68.64 ± 14.13	68.75 ± 13.97	65.27 ± 16.33	70.18 ± 12.73	69.72 ± 13.40	<0.001
SBP, mmHg	130.18 ± 22.84	127.82 ± 22.51	125.43 ± 22.73	134.14 ± 21.12	136.19 ± 23.87	<0.001
DBP, mmHg	76.84 ± 15.90	76.34 ± 16.04	76.03 ± 14.99	78.41 ± 15.68	77.02 ± 16.53	0.117
HR, bpm	82.63 ± 20.72	80.49 ± 20.34	85.87 ± 21.63	82.64 ± 20.66	85.14 ± 20.29	<0.001
BMI, kg/m^2^	24.29 ± 3.78	24.16 ± 3.88	24.13 ± 3.52	24.36 ± 3.80	24.72 ± 3.69	0.105
BNP, pg./mL	574.65 (214.08, 1159.25)	547.00 (214.17, 1109.96)	526.50 (197.12, 1107.50)	700.00 (256.00, 1350.00)	542.50 (210.24, 1205.28)	0.043
Hb, g/L	126.11 ± 23.14	128.69 ± 21.96	124.71 ± 24.56	124.48 ± 22.11	122.67 ± 25.24	<0.001
ALB, g/L	37.81 ± 4.00	38.17 ± 4.02	37.36 ± 4.53	37.86 ± 3.35	37.26 ± 4.02	<0.001
TG, mg/dL	89.43 (68.18, 124.84)	84.11 (63.61, 107.85)	128.83 (90.31, 188.81)	76.14 (59.32, 93.48)	125.99 (89.84, 180.84)	<0.001
TC, mg/dL	141.92 (116.01, 172.47)	142.69 (117.68, 172.47)	154.31 (122.09, 188.71)	128.77 (109.44, 154.68)	146.98 (118.17, 178.48)	<0.001
HDL-C, mg/dL	38.67 (31.71, 47.56)	41.38 (34.42, 51.04)	34.03 (27.46, 42.92)	38.67 (32.87, 49.11)	35.96 (30.16, 40.99)	<0.001
LDL-C, mg/dL	78.11 (58.01, 104.41)	79.27 (59.94, 104.80)	87.78 (62.26, 116.01)	70.38 (52.20, 88.55)	80.43 (56.07, 114.08)	<0.001
eGFR, mL/min/1.73 m^2^	68.78 ± 28.06	72.83 ± 25.16	71.79 ± 31.34	65.08 ± 27.85	59.69 ± 29.59	<0.001
UA, μmol/L	428.00 (338.65, 533.62)	413.00 (328.18, 516.00)	449.90 (352.00, 557.00)	424.00 (341.50, 531.50)	435.80 (351.25, 560.75)	0.012
FPG, mg/dL	90.75 (81.36, 111.47)	84.70 (78.12, 92.43)	95.76 (86.89, 108.95)	96.12 (81.72, 117.35)	138.48 (111.24, 180.81)	<0.001
HbA1c, %	6.45 ± 1.28	5.80 ± 0.48	5.97 ± 0.77	7.20 ± 1.29	7.72 ± 1.62	<0.001
CRP, mg/L	4.50 (3.00, 10.93)	4.30 (2.90, 10.36)	5.34 (3.38, 12.00)	4.80 (3.00, 12.00)	4.46 (2.80, 8.93)	0.010
CTI	9.12 (8.63, 9.69)	8.79 (8.44, 9.09)	9.88 (9.67, 10.22)	8.87 (8.39, 9.19)	10.03 (9.73, 10.48)	<0.001
LVEF, %	42.59 ± 11.35	42.35 ± 11.47	43.19 ± 11.35	42.17 ± 11.47	43.14 ± 10.91	0.434
LAD, cm	4.60 (4.00, 5.16)	4.70 (4.07, 5.20)	4.40 (3.60, 5.01)	4.64 (4.20, 5.21)	4.57 (4.03, 4.95)	<0.001
LVDd, cm	5.80 (5.15, 6.50)	5.83 (5.10, 6.65)	5.80 (5.10, 6.60)	5.80 (5.17, 6.50)	5.75 (5.26, 6.40)	0.791
Coronary artery disease, *n* (%)	468 (23.4)	241 (26.6)	80 (24.1)	94 (22.5)	53 (15.4)	<0.001
Atrial fibrillation, *n* (%)	880 (44.0)	444 (49.0)	127 (38.3)	173 (41.5)	136 (39.5)	<0.001
Hypertension, *n* (%)	1,225 (61.3)	474 (52.3)	168 (50.6)	314 (75.3)	269 (78.2)	<0.001
Chronic obstructive pulmonary disease, *n* (%)	87 (4.3)	47 (5.2)	12 (3.6)	13 (3.1)	15 (4.4)	0.326
Statin use, *n* (%)	1,112 (55.6)	459 (50.6)	174 (52.4)	250 (60.0)	229 (66.6)	<0.001
Antiplatelet agent use, *n* (%)	1,040 (52.0)	443 (48.8)	169 (50.9)	221 (53.0)	207 (60.2)	0.004
MRA use, *n* (%)	1,091 (54.5)	518 (57.1)	210 (63.3)	200 (48.0)	163 (47.4)	<0.001
Beta-blocker use, *n* (%)	1,533 (76.6)	678 (74.8)	255 (76.8)	327 (78.4)	273 (79.4)	0.265
Diuretic use, *n* (%)	1723 (86.2)	772 (85.1)	292 (88.0)	364 (87.3)	295 (85.8)	0.529
ACEI/ARB/ARNI use, *n* (%)	944 (47.2)	412 (45.4)	177 (53.3)	200 (48.0)	155 (45.1)	0.077
SGLT2 inhibitor use, *n* (%)	342 (17.1)	110 (12.1)	43 (13.0)	91 (21.8)	98 (28.5)	<0.001

SBP, systolic blood pressure; DBP, diastolic blood pressure; HR, heart rate; BMI, body mass index; BNP, B-type natriuretic peptide; Hb, haemoglobin; ALB, albumin; TC, total cholesterol; TG, triglycerides; HDL-C, high-density lipoprotein cholesterol; LDL-C, low-density lipoprotein cholesterol; FPG, fasting plasma glucose; HbA1c, glycated haemoglobin; eGFR, estimated glomerular filtration rate; UA, uric acid; CRP, C-reactive protein; CTI, C-reactive protein–triglyceride–glucose index; DM, diabetes mellitus; LVEF, left ventricular ejection fraction; LVDd, left ventricular end-diastolic diameter; LAD, left atrial diameter; MRA, mineralocorticoid receptor antagonist; ACEI, angiotensin-converting enzyme inhibitor; ARB, angiotensin receptor blocker; ARNI, angiotensin receptor–neprilysin inhibitor; SGLT2i, sodium–glucose cotransporter 2 inhibitor.

The distribution of qualifying CKM-related abnormalities is shown in Supplementary Table S11. Most patients had multiple non-cardiovascular CKM-related abnormalities, with 72.6% having a CKM burden of ≥3. This pattern supports the interpretation of the cohort as an ADHF population embedded within a broader CKM context rather than cardiovascular disease alone.

### Associations of diabetes status and CTI level with the primary outcomes

Cox regression results for the four groups defined by diabetes status and CTI level are presented in Table 2. In the fully adjusted model, compared with the non-DM + low CTI group, the non-DM + high CTI group was associated with higher risks of all-cause mortality (HR 1.54, 95% CI 1.15–2.06, *p* = 0.004) and MACCEs (HR 1.37, 95% CI 1.08–1.75, *p* = 0.011), whereas the DM + low CTI group was not significantly associated with either outcome. The DM + high CTI group had the highest risk, with adjusted HRs of 1.95 (95% CI 1.47–2.58, *p* < 0.001) for all-cause mortality and 1.91 (95% CI 1.51–2.41, *p* < 0.001) for MACCEs.

**Table 2:** Associations of diabetes status and CTI level with all-cause mortality and MACCEs.

Group	Events/Total	Model 1		Model 2		Model 3	
		HR (95% CI)	*p*	HR (95% CI)	*p*	HR (95% CI)	*p*
All-cause mortality							
non-DM + low CTI	139/907	1.00 (reference)	—	1.00 (reference)	—	1.00 (reference)	—
non-DM + high CTI	72/332	1.54 (1.16–2.05)	0.003	1.72 (1.29–2.29)	<0.001	1.54 (1.15–2.06)	0.004
DM + low CTI	61/417	0.97 (0.72–1.31)	0.854	0.95 (0.70–1.28)	0.738	0.82 (0.60–1.13)	0.228
DM + high CTI	94/344	2.02 (1.55–2.62)	<0.001	2.00 (1.54–2.60)	<0.001	1.95 (1.47–2.58)	<0.001
MACCEs							
non-DM + low CTI	208/907	1.00 (reference)	—	1.00 (reference)	—	1.00 (reference)	—
non-DM + high CTI	102/332	1.43 (1.13–1.81)	0.003	1.49 (1.18–1.90)	<0.001	1.37 (1.08–1.75)	0.011
DM + low CTI	111/417	1.18 (0.94–1.49)	0.152	1.14 (0.91–1.44)	0.256	1.10 (0.87–1.40)	0.413
DM + high CTI	130/344	1.93 (1.55–2.40)	<0.001	1.94 (1.55–2.41)	<0.001	1.91 (1.51–2.41)	<0.001

CTI, C-reactive protein-triglyceride-glucose index; DM, diabetes mellitus; MACCEs, major adverse cardiac and cerebrovascular events; HR, hazard ratio; CI, confidence interval.

Model 1: Unadjusted.

Model 2: Adjusted for age and sex.

Model 3: Adjusted for age, sex, BMI, SBP, HR, eGFR, LVEF, BNP, atrial fibrillation, coronary artery disease, and the use of SGLT2 inhibitors, ACEIs/ARBs/ARNIs, beta-blockers, MRAs, diuretics, and statins.

**Figure 2:**
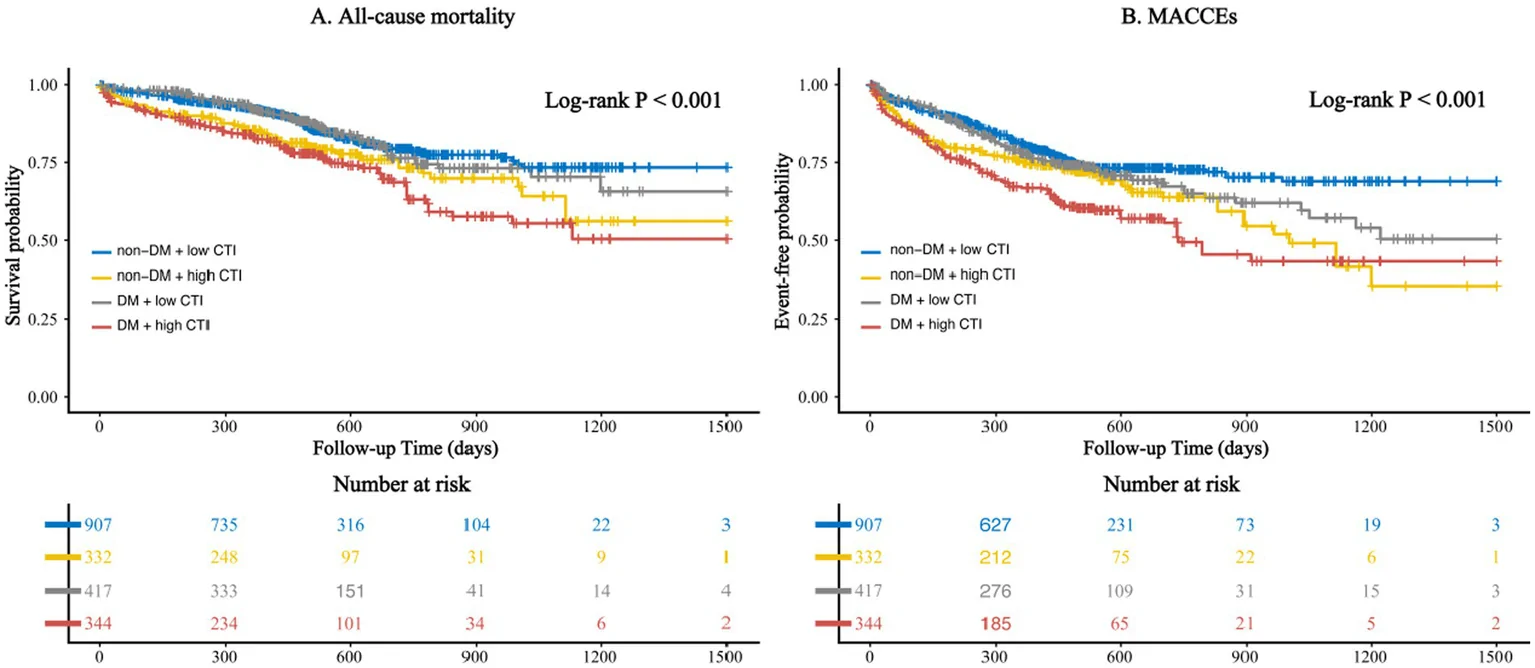
Conventional Kaplan–Meier curves according to diabetes status and CTI level. Kaplan–Meier curves are shown for all-cause mortality **(A)** and major adverse cardiac and cerebrovascular events (MACCEs) **(B)** according to the four groups defined by diabetes status and CTI level: non-DM + low CTI, non-DM + high CTI, DM + low CTI, and DM + high CTI. Numbers at risk are shown below the x-axis. *p* values were calculated using the log-rank test. CTI, C-reactive protein–triglyceride–glucose index; DM, diabetes mellitus; MACCEs, major adverse cardiac and cerebrovascular events.

Conventional Kaplan–Meier curves demonstrated significant crude separation across the four groups for both all-cause mortality and MACCEs (Figure 2). The DM + high CTI group showed the lowest survival and event-free probability, whereas the non-DM + low CTI group had the most favourable outcomes. The non-DM + high CTI group also showed worse event-free survival than the non-DM + low CTI group, while the DM + low CTI group showed a crude survival pattern closer to that of the non-DM + low CTI group, particularly for all-cause mortality. Group differences were significant for both outcomes by the log-rank test.

**Figure 3:**
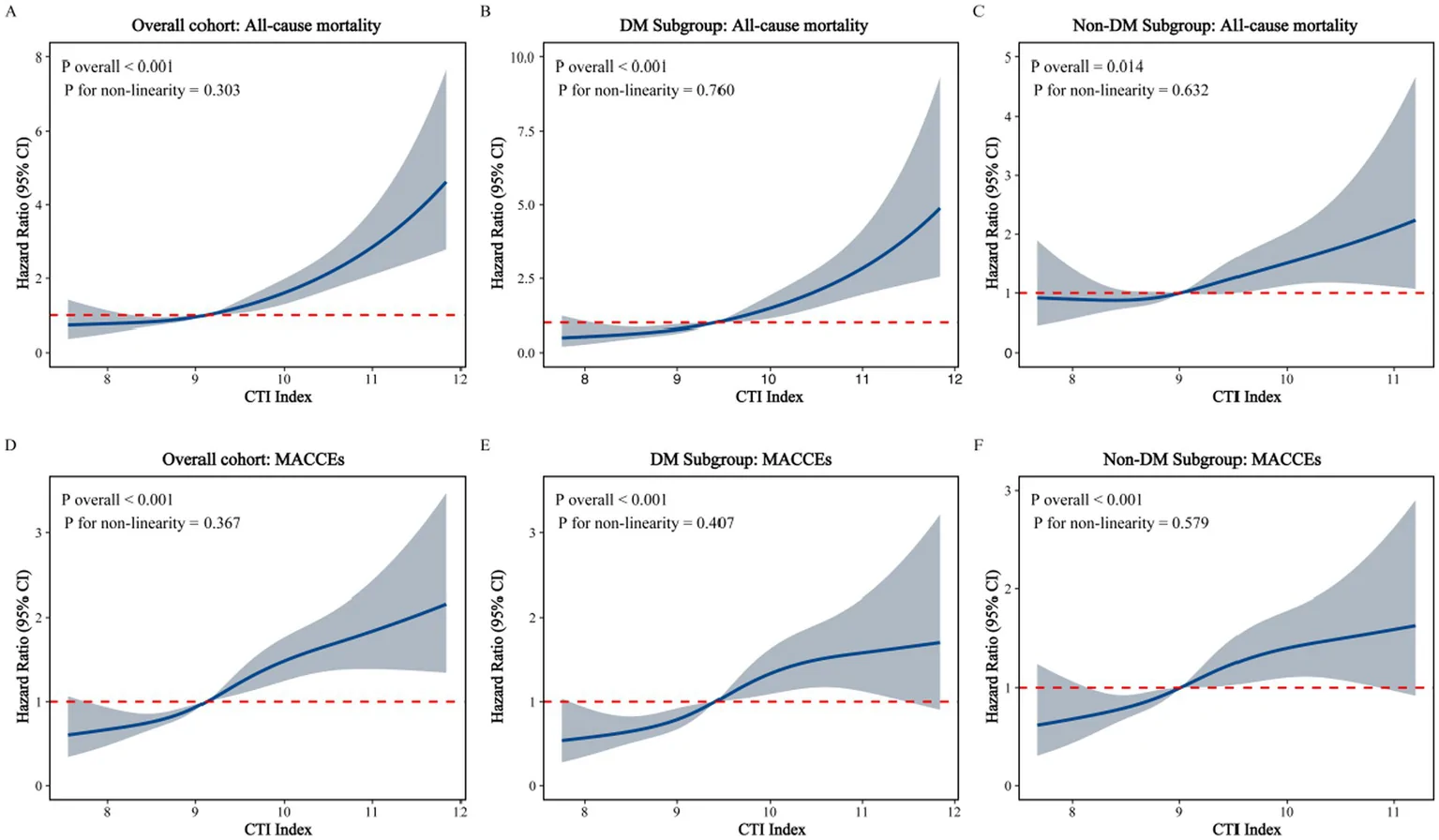
Restricted cubic spline analyses of the associations between CTI and all-cause mortality and MACCEs in the overall cohort and in the DM and non-DM subgroups. **(A–C)** Associations between CTI and all-cause mortality in the overall cohort, DM subgroup, and non-DM subgroup, respectively. **(D–F)** Associations between CTI and MACCEs in the overall cohort, DM subgroup, and non-DM subgroup, respectively. Solid lines indicate hazard ratios, and shaded areas indicate 95% confidence intervals. *p* values for overall association and non-linearity are shown in each panel.

Restricted cubic spline analyses showed significant and approximately linear associations between CTI and both all-cause mortality and MACCEs in the overall population (all P for overall < 0.05; all P for non-linearity > 0.05), with similar patterns in both non-DM and DM subgroups (Figure 3). Consistently, when analysed as a continuous variable, CTI remained significantly associated with both outcomes in the non-DM and DM subgroups (Supplementary Table S2). No significant interaction was observed between diabetes status and CTI, whether CTI was modelled as a continuous variable or as a high versus low categorical variable (Supplementary Tables S3, S4).

In supplementary analyses using the three AHA glycaemic categories rather than the primary classification based on prior diagnosis of DM, Kaplan–Meier analyses based on glycaemic categories alone showed significant differences for MACCEs but not for all-cause mortality (Supplementary Figure S1). When CTI was modelled continuously across the three glycaemic categories, it was associated with MACCEs in all categories, whereas its association with all-cause mortality was mainly evident in the prediabetes and diabetes groups. Similar patterns were observed for high versus low CTI, with no significant interaction by glycaemic category for either outcome (Supplementary Table S5). Restricted cubic spline analyses showed a broadly similar overall pattern, with more evident associations in the prediabetes and diabetes groups (Supplementary Figure S2).

### Incremental prognostic value of CTI compared with related inflammatory-metabolic biomarker models

To determine whether CTI provided prognostic information beyond diabetes status and related inflammatory and metabolic biomarkers, we compared a series of biomarker models using the clinical model plus diabetes status as the reference model (Table 3). For all-cause mortality, adding CTI increased the C-index from 0.656 to 0.674 (Δ = 0.018) and the 1-year time-dependent AUC from 0.666 to 0.689 (Δ = 0.023), representing the largest gains among the evaluated biomarker models. Nevertheless, the absolute improvements were modest, and the gains with TyG were smaller. For MACCEs, adding CTI increased the C-index from 0.623 to 0.639 (Δ = 0.016) and the 1-year time-dependent AUC from 0.641 to 0.647 (Δ = 0.006), again representing the largest gains among the evaluated biomarker models. These improvements were also modest, particularly for the 1-year time-dependent AUC, for which TyG produced no improvement. The model based on the four diabetes/CTI groups also improved performance for both outcomes. Other performance metrics, including likelihood-ratio tests, reclassification indices, prediction error, and calibration slope, are presented in Table 3.

**Table 3:** Incremental prognostic value of CTI compared with related inflammatory and metabolic biomarker models.

Outcomes and Model	C-index	ΔC-index	1-year tdAUC	ΔtdAUC	LR test P	Continuous NRI	P for NRI	IDI	P for IDI	Brier score	Calibration slope
All-cause mortality											
Clinical model + DM	0.656	—	0.666	—	—	—	—	—	—	0.090	1.136
+ CRP	0.657	0.001	0.665	−0.001	0.037	0.014	0.813	0.002	0.222	0.089	1.104
+ TG	0.659	0.003	0.670	0.004	0.047	0.191	0.011	0.002	0.014	0.089	1.135
+ FPG	0.662	0.006	0.672	0.006	<0.001	0.296	<0.001	0.014	<0.001	0.088	1.119
+ TyG	0.664	0.008	0.678	0.012	<0.001	0.282	<0.001	0.012	<0.001	0.088	1.131
+ CRP + TyG	0.665	0.009	0.677	0.011	<0.001	0.304	<0.001	0.015	<0.001	0.088	1.117
+ CRP + TG + FPG	0.664	0.008	0.674	0.008	<0.001	0.254	<0.001	0.017	<0.001	0.088	1.102
+ CTI	0.674	0.018	0.689	0.023	<0.001	0.437	<0.001	0.029	<0.001	0.087	1.079
Clinical model + diabetes/CTI classification	0.669	0.013	0.686	0.020	<0.001	0.407	<0.001	0.022	<0.001	0.087	1.118
MACCEs											
Clinical model + DM	0.623	—	0.641	—	—	—	—	—	—	0.171	1.081
+ CRP	0.622	−0.001	0.643	0.002	0.083	0.008	0.846	0.003	0.006	0.170	1.091
+ TG	0.623	0.000	0.641	0.000	0.772	0.062	0.268	0.000	0.597	0.171	1.080
+ FPG	0.623	0.000	0.637	−0.004	0.002	0.177	0.002	0.007	0.002	0.170	1.029
+ TyG	0.624	0.001	0.638	−0.003	0.004	0.087	0.124	0.005	0.004	0.170	1.036
+ CRP + TyG	0.623	0.000	0.640	−0.001	0.006	0.107	0.046	0.007	<0.001	0.169	1.061
+ CRP + TG + FPG	0.622	−0.001	0.639	−0.002	0.007	0.167	0.004	0.009	<0.001	0.169	1.037
+ CTI	0.639	0.016	0.647	0.006	<0.001	0.293	<0.001	0.018	<0.001	0.168	0.983
Clinical model + diabetes/CTI classification	0.632	0.009	0.645	0.004	<0.001	0.230	<0.001	0.012	<0.001	0.169	1.026

Values are shown for the clinical model plus diabetes status and for models additionally incorporating untransformed individual biomarkers, TyG, CRP + TyG, CRP + TG + FPG, CTI, or the diabetes/CTI classification. The clinical model included age, sex, BMI, systolic blood pressure, heart rate, eGFR, LVEF, BNP, atrial fibrillation, coronary artery disease, and baseline cardiovascular medications (SGLT2 inhibitor, ACEI/ARB/ARNI, beta-blocker, MRA, diuretic, and statin use). The diabetes/CTI classification model included the same clinical covariates and the four-category diabetes/CTI grouping variable, without diabetes status entered separately. ΔC-index and ΔtdAUC are relative to the Clinical model + DM model within each outcome. Prediction metrics, including 1-year tdAUC, Brier score, calibration slope, IDI, and continuous NRI, were evaluated at the prespecified 1-year prediction horizon. A lower Brier score indicates better overall prediction accuracy. CTI, C-reactive protein–triglyceride–glucose index; CRP, C-reactive protein; DM, diabetes mellitus; FPG, fasting plasma glucose; IDI, integrated discrimination improvement; LR, likelihood-ratio; MACCEs, major adverse cardiac and cerebrovascular events; NRI, net reclassification improvement; tdAUC, time-dependent area under the curve; TG, triglycerides; TyG, triglyceride–glucose index.

Calibration and decision curve analyses at the 1-year prediction horizon showed acceptable calibration and broadly comparable net benefit across models, with modest support for the CTI model across selected threshold probability ranges (Supplementary Figure S5).

### Subgroup analyses

**Figure 4:**
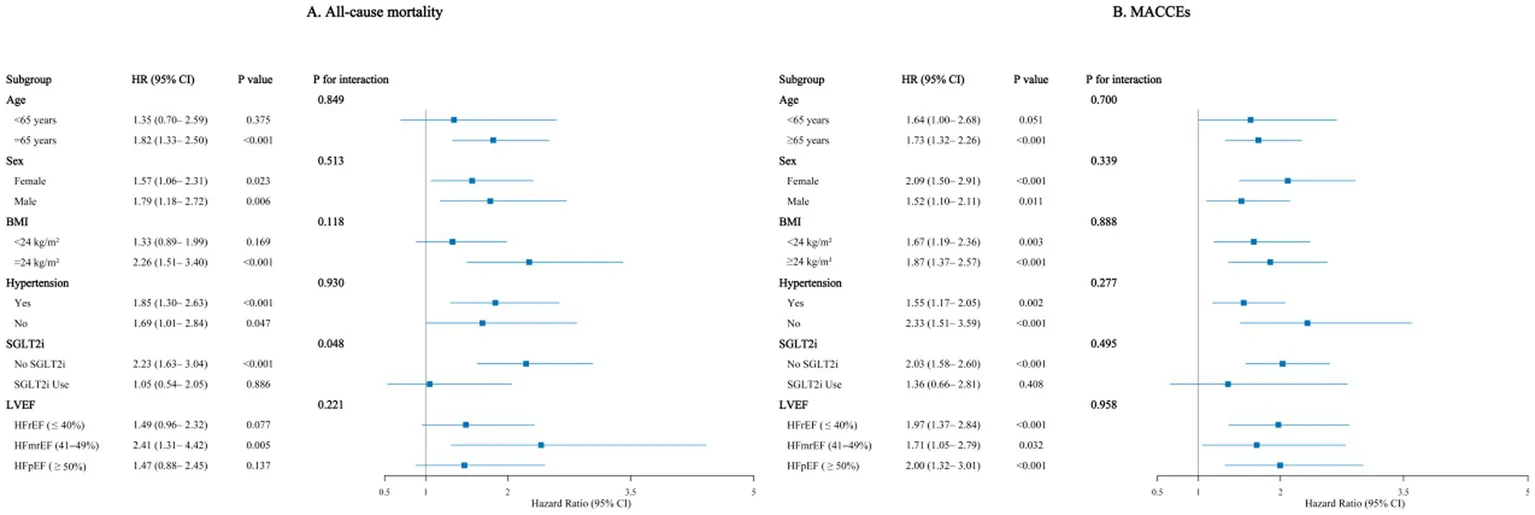
Forest plots for subgroup analyses of the associations of the DM + high CTI group with all-cause mortality and MACCEs. **(A)** All-cause mortality. **(B)** Major adverse cardiac and cerebrovascular events (MACCEs). Hazard ratios (HRs) and 95% confidence intervals (CIs) are shown for the association of the DM + high CTI group with study outcomes across clinically relevant subgroups, using the non-DM + low CTI group as the reference group. Nominal interaction *p* values are shown; FDR-adjusted values for the 24-test family are reported in Supplementary Table S12.

Subgroup associations for the DM + high CTI and non-DM + high CTI groups are presented in Figure 4 and Supplementary Figure S4, respectively. None of the 24 interaction tests remained statistically significant after Benjamini–Hochberg FDR correction (Supplementary Table S12). The nominal interaction with SGLT2 inhibitor use for all-cause mortality (P for interaction = 0.048) was no longer significant after correction (FDR-adjusted *p* = 0.651). Given the small number of patients receiving SGLT2 inhibitors (*n* = 342) and the imprecision of the subgroup estimates (Supplementary Table S6), these findings were considered exploratory. Corresponding descriptive Kaplan–Meier curves stratified by SGLT2 inhibitor use are shown in Supplementary Figure S3.

### Sensitivity analyses

Competing-risk, 30-day landmark, stratified Cox, and inflammatory-marker exclusion analyses broadly supported the primary findings (Supplementary Tables S7–S10). The complete-case analysis included 1,588 patients (79.4%), among whom 297 deaths and 433 MACCEs occurred. In this analysis, DM + high CTI remained associated with all-cause mortality (HR 1.94, 95% CI 1.42–2.66; *p* < 0.001) and MACCEs (HR 1.85, 95% CI 1.42–2.40; *p* < 0.001). Non-DM + high CTI also remained associated with all-cause mortality (HR 1.65, 95% CI 1.20–2.27; *p* = 0.002), whereas its association with MACCEs was attenuated and no longer statistically significant (HR 1.31, 95% CI 0.99–1.73; *p* = 0.054) (Supplementary Table S13).

## Discussion

In this single-centre retrospective cohort of patients hospitalized for ADHF with CKM stage 4, diabetes status and CTI level jointly stratified the risks of all-cause mortality and MACCEs. Several findings warrant emphasis. First, elevated CTI was associated with higher risks of both outcomes, including among patients without documented diabetes. Second, diabetes alone did not uniformly identify higher-risk patients, because the DM + low CTI group was not significantly associated with either outcome after multivariable adjustment, whereas the DM + high CTI group had the worst prognosis. Third, CTI showed approximately linear associations with all-cause mortality and MACCEs, and no significant interaction was observed between CTI and diabetes status, suggesting that the prognostic association of CTI was broadly consistent across diabetes strata. Fourth, compared with CRP, TG, FPG, TyG, CRP + TyG, and the combined CRP + TG + FPG component model, CTI provided greater, although modest, incremental prognostic information when added to the clinical model plus diabetes status. Sensitivity analyses broadly supported the primary findings. Taken together, these findings support further evaluation of CTI as a candidate adjunctive marker for risk refinement in patients hospitalized for ADHF with CKM stage 4.

From a metabolism-focused perspective, CTI may be viewed as an admission-period composite inflammatory-metabolic marker rather than a heart failure-specific biomarker or a stable metabolic trait. Its components reflect glycaemic status, triglyceride-related metabolic disturbance, and systemic inflammation, which are closely related to metabolic health, insulin resistance, adiposity-related risk, and CKM progression. In this context, the present findings suggest that inflammatory-metabolic risk is not fully captured by diabetes status alone, even in patients with established cardiovascular disease and advanced CKM syndrome.

These findings are consistent with the CKM framework, in which established cardiovascular disease occurs in the context of metabolic, renal, and adiposity-related abnormalities (9). In patients hospitalized for ADHF, prognosis may be influenced not only by haemodynamic impairment, but also by metabolic stress, systemic inflammation, renal dysfunction, neurohormonal activation, adipose tissue dysfunction, and cardiorenal interactions (17). CTI combines routinely available information from fasting plasma glucose, triglycerides, and C-reactive protein, representing glycaemic, lipid-related, and inflammatory domains that may not be fully captured by conventional clinical risk markers (8, 18–22).

The observed group pattern is clinically informative. However, the lack of statistical significance for the DM + low CTI group should not be taken to indicate a risk equivalent to that of the non-DM + low CTI reference group, adequate glycaemic control, or protection against adverse outcomes. Because data on diabetes duration, longitudinal glycaemic control, treatment adherence, and post-discharge treatment changes were unavailable, we could not determine whether the observed risk pattern was related to differences in diabetes severity, metabolic control, or treatment. Accordingly, this result indicates only that the association did not reach statistical significance and does not identify a distinct lower-risk diabetes phenotype. Conversely, the adverse prognosis observed in the non-DM + high CTI group indicates that the absence of documented diabetes does not imply metabolic stability. Patients without documented diabetes may still have insulin resistance, prediabetes, stress-related hyperglycaemia, adipose tissue dysfunction, or inflammatory activation (11, 16). In this regard, CTI may complement diabetes status by identifying marker-defined risk differences that would be obscured by a diabetes-only classification. Because CTI is a composite marker, a high CTI value may arise from different combinations of inflammatory and metabolic abnormalities across patients and should not be interpreted as a uniform biological phenotype. The lack of significant interaction between CTI and diabetes status suggests that, despite potential differences in component composition across diabetes strata, the prognostic association of CTI was broadly consistent. Accordingly, the four categories should be interpreted as descriptive joint risk strata rather than as evidence of effect modification (8, 19, 20, 23).

The association between elevated CTI and adverse outcomes is biologically plausible but should be interpreted cautiously. CTI combines CRP, triglycerides, and fasting plasma glucose, which may represent overlapping clinical signals of systemic inflammation, lipid-related metabolic disturbance, and glycaemic stress during ADHF hospitalization. The modestly better prognostic performance of CTI than its individual components may reflect the integration of these partially complementary signals; however, this interpretation remains speculative and was not tested mechanistically. Previous experimental and translational studies have linked these domains to endothelial dysfunction, impaired myocardial substrate handling, oxidative stress, renal dysfunction, and adverse cardiac remodelling. Inflammatory pathways, including NF-κB signalling and NLRP3 inflammasome activation, may also contribute to inflammatory-metabolic crosstalk in heart failure. However, the present study cannot determine whether CTI reflects a causal pathway, a stable biological phenotype, or acute illness severity. This distinction is particularly important in ADHF, where haemodynamic instability, congestion, adrenergic activation, inflammatory responses, renal dysfunction, treatment initiation, and stress hyperglycaemia may influence CRP, triglycerides, and glucose in different directions. Therefore, CTI should be interpreted primarily as a composite risk marker rather than as direct evidence of a specific mechanistic pathway (24–31).

Our findings are broadly consistent with previous studies showing that CTI, TyG-related indices, AIP, and other integrated metabolic markers are associated with cardiovascular events and mortality in general populations, diabetic cohorts, and individuals with CKM stages 0–3. However, the present study extends this literature by shifting the focus from early risk detection to risk refinement within advanced CKM syndrome. Most prior studies have examined earlier CKM stages, incident cardiovascular disease, or community-based populations, whereas patients with CKM stage 4 hospitalized for ADHF already represent a clinically high-risk group in whom additional risk discrimination is more challenging. Nevertheless, CTI remained prognostically informative despite the high baseline risk. These findings suggest that outcome heterogeneity among patients with CKM stage 4 heart failure is not fully explained by CKM stage or diabetes status alone, and that CTI may provide additional risk-stratification information (11–14, 32).

Adding CTI to the clinical model plus diabetes status yielded modest improvements in discrimination for all-cause mortality (ΔC-index = 0.018; Δ1-year time-dependent AUC = 0.023) and MACCEs (ΔC-index = 0.016; Δ1-year time-dependent AUC = 0.006), although these gains were larger than those obtained with the evaluated comparator biomarker models. Such modest improvements are not unexpected in this high-risk ADHF cohort because the reference model already included strong prognostic determinants. In this setting, reclassification metrics may provide complementary information on changes in individual risk classification (33–36). The observed improvement in reclassification suggests that CTI adds prognostic information beyond diabetes status and established clinical predictors. Calibration was broadly comparable across models, and decision-curve analysis showed only limited differences in net benefit. These findings do not establish improved clinical decision-making or patient outcomes; therefore, CTI should be regarded as an adjunctive marker for risk refinement rather than a standalone prediction tool (35–39).

In exploratory analyses stratified by SGLT2 inhibitor use, DM + high CTI was no longer significantly associated with all-cause mortality among users, whereas non-DM + high CTI remained associated with higher mortality. Among patients not receiving SGLT2 inhibitors, both high-CTI groups were associated with higher mortality. However, the nominal interaction for all-cause mortality did not remain statistically significant after FDR correction, and none of the subgroup interaction tests remained significant after correction. Given the limited subgroup size, nonrandomized treatment allocation, imprecise estimates, and potential confounding by indication, these findings should be interpreted as hypothesis-generating and exploratory rather than as evidence that SGLT2 inhibitors modify the risk associated with elevated CTI (40–44).

From a clinical standpoint, CTI is not yet a validated decision tool. The components required to calculate CTI are inexpensive, routinely available, and easily standardized; however, this study did not establish a clinically actionable threshold or evaluate whether CTI-guided management improves treatment allocation or patient outcomes. Therefore, the findings do not establish that a high CTI should trigger a specific change in management. CTI should not replace established prognostic markers such as natriuretic peptides, conventional prediction models, or guideline-directed clinical assessment. Its potential role is that of a candidate adjunctive marker requiring external validation and prospective evaluation of clinical utility (2, 18, 40–42).

## Limitations

Several limitations should be considered. First, this retrospective single-centre study from a Chinese inpatient cohort may introduce selection bias, limit generalisability, and preclude causal inference. Excluding 988 of 2,988 screened patients, including those without calculable CTI or follow-up, may have selected a more completely characterized cohort. CKM stage 4 was operationally defined using retrospective inpatient data, and because ADHF commonly coexists with metabolic, renal, and adiposity-related abnormalities, the present cohort inevitably overlaps with contemporary real-world ADHF populations. Therefore, our findings should be interpreted as risk refinement within an ADHF population meeting CKM stage 4 criteria rather than as evidence of a distinct heart failure phenotype. Because the qualifying CKM components were ascertained from routinely collected inpatient records, incomplete documentation may also have caused staging misclassification. BMI may also be influenced by congestion, fluid retention, and decongestive therapy in ADHF, so misclassification of adiposity-related CKM abnormalities cannot be excluded. Second, residual confounding remains possible despite multivariable adjustment and sensitivity analyses, particularly from unmeasured factors such as diabetes duration, frailty, congestion severity, and post-discharge treatment changes. Diabetes status was based on documented prior diagnosis, although supplementary analyses using FPG, HbA1c, and diabetes history yielded broadly consistent findings. Supplementary glycaemic reclassification does not eliminate possible misclassification of undiagnosed diabetes. The model was prognostic rather than causal, and the unverifiable MAR assumption leaves potential for missing-data bias. Third, CTI was measured during the index hospitalization and may partly reflect acute illness severity rather than stable chronic risk; serial measurements are needed to evaluate the prognostic value of persistent elevation or dynamic changes in CTI. The 30-day landmark analysis was restricted to patients who remained alive and free of the relevant outcome at 30 days and therefore cannot eliminate acute-phase measurement bias or survivor selection. Fourth, the cohort-derived 9.50 threshold requires external validation. The MACCE composite included hospitalization for worsening heart failure, which is sensitive to healthcare access and admission practices and may limit cross-system comparisons. Finally, the modest discrimination gains and unproven clinical actionability require external validation before clinical use.

## Conclusion

In conclusion, among patients with stage 4 CKM syndrome hospitalized for acute decompensated heart failure, elevated CTI was associated with higher risks of all-cause mortality and MACCEs, with the greatest risk observed in patients with both diabetes and high CTI. CTI provided greater, although modest, incremental prognostic information than the evaluated inflammatory-metabolic biomarkers when added to the clinical model plus diabetes status. These findings support further evaluation of CTI as a candidate adjunctive risk marker rather than a clinical decision tool. External validation in independent and more diverse cohorts and prospective evaluation of clinical utility are required before routine use.

## Data Availability

The data used in this study were obtained from the hospital information system. Because of patient privacy restrictions, the datasets are not publicly available. Data may be obtained from the corresponding author upon reasonable request.

## Glossary

ADHF: acute decompensated heart failure
AF: atrial fibrillation
AHA: American Heart Association
CAD: coronary artery disease
CKD: chronic kidney disease
CKM: cardiovascular–kidney–metabolic
CVD: cardiovascular disease
DM: diabetes mellitus
HF: heart failure
MACCEs: major adverse cardiac and cerebrovascular events
MI: myocardial infarction
AIP: atherogenic index of plasma
ALB: albumin
ALT: alanine aminotransferase
AST: aspartate aminotransferase
BNP: B-type natriuretic peptide
CRP: C-reactive protein
CTI: C-reactive protein–triglyceride–glucose index
eGFR: estimated glomerular filtration rate
FPG: fasting plasma glucose
Hb: haemoglobin
HbA1c: glycated haemoglobin A1c
HDL-C: high-density lipoprotein cholesterol
LDL-C: low-density lipoprotein cholesterol
TC: total cholesterol
TG: triglycerides
TyG: triglyceride–glucose index
TyG-BMI: triglyceride–glucose–body mass index
UA: uric acid
WBC: white blood cell count
BMI: body mass index
DBP: diastolic blood pressure
HR: heart rate in baseline characteristics and hazard ratio in survival analyses
LAD: left atrial diameter
LVDd: left ventricular end-diastolic diameter
LVEF: left ventricular ejection fraction
SBP: systolic blood pressure
ACEI: angiotensin-converting enzyme inhibitor
ARB: angiotensin receptor blocker
ARNI: angiotensin receptor–neprilysin inhibitor
MRA: mineralocorticoid receptor antagonist
SGLT2i: sodium–glucose cotransporter 2 inhibitor
CI: confidence interval
DCA: decision curve analysis
IDI: integrated discrimination improvement
IQR: interquartile range
LR test: likelihood-ratio test
NRI: net reclassification improvement
RCS: restricted cubic spline
SD: standard deviation
NF-κB: nuclear factor kappa B
NLRP3: NOD-like receptor family pyrin domain-containing 3
