# Supplementary material for: C-reactive protein–triglyceride–glucose index and diabetes status for risk stratification in stage 4 cardiovascular–kidney–metabolic syndrome with acute decompensated heart failure: a retrospective cohort study
Source: Front Nutr. 2026 Sep 9;13:1903670. doi: 10.3389/fnut.2026.1903670 (PMC13597441; doi:10.3389/fnut.2026.1903670)
Supplement: Supplementary file 1 [file Supplementary_file_1.DOCX]

Figure S1. Kaplan–Meier curves for all-cause mortality and MACCEs according to glycaemic status.

(A) All-cause mortality.

(B) Major adverse cardiac and cerebrovascular events (MACCEs).

Patients were categorized into three glycaemic groups: normoglycaemia, prediabetes, and diabetes. Numbers at risk are shown below the x-axis.

Figure S2. Restricted cubic spline analyses of the associations between CTI and clinical outcomes according to glycaemic status.

(A–C) Associations between CTI and all-cause mortality in the normoglycaemia, prediabetes, and diabetes groups, respectively.

(D–F) Associations between CTI and MACCEs in the normoglycaemia, prediabetes, and diabetes groups, respectively.

Solid blue lines indicate hazard ratios, shaded areas indicate 95% confidence intervals, and red dashed lines indicate the reference hazard ratio of 1. P values for overall association and non-linearity are shown in each panel.

Figure S3. Kaplan–Meier curves for all-cause mortality according to diabetes status and CTI level, stratified by SGLT2 inhibitor use.

(A) Patients not receiving SGLT2 inhibitors.

(B) Patients receiving SGLT2 inhibitors.

Patients were classified into four groups according to diabetes status and CTI level: non-DM + low CTI, non-DM + high CTI, DM + low CTI, and DM + high CTI. P values were derived from the log-rank test, and numbers at risk are shown below the x-axis.

**Figure S4. Subgroup analyses of the associations of the non-DM + high CTI group with all-cause mortality and MACCEs.**

Hazard ratios (HRs) and 95% confidence intervals (CIs) are shown for the association of the non-DM + high CTI group with study outcomes across clinically relevant subgroups, using the non-DM + low CTI group as the reference group. Nominal interaction P values are shown; FDR-adjusted values for the 24-test family are reported in Table S12.

**Figure S5. Calibration and decision curve analyses for 1-year outcomes.**

(A, C) Calibration curves for 1-year all-cause mortality (A) and MACCEs (C). Predicted risks were grouped into quintiles, and observed risks were estimated using the Kaplan–Meier method. The dashed diagonal line represents perfect calibration.

(B, D) Decision curve analyses for 1-year all-cause mortality (B) and MACCEs (D). Net benefit was calculated across clinically relevant threshold probabilities. The grey dashed and dotted lines indicate the treat-all and treat-none strategies, respectively.

The components model included the clinical model plus diabetes status, with CRP, TG, and FPG entered as separate terms.
