# Supplementary material for: C-reactive protein–triglyceride–glucose index and diabetes status for risk stratification in stage 4 cardiovascular–kidney–metabolic syndrome with acute decompensated heart failure: a retrospective cohort study
Source: Front Nutr. 2026 Sep 9;13:1903670. doi: 10.3389/fnut.2026.1903670 (PMC13597441; doi:10.3389/fnut.2026.1903670)
Supplement: Supplementary file 2 [file Table_1.DOCX]

**Table S1. Baseline characteristics of patients with acute decompensated heart failure within CKM stage 4 according to AHA glycaemic categories.**

| **Characteristic** | **Overall** | **Normoglycaemia** | **Prediabetes** | **Diabetes** | **P value** |
| --- | --- | --- | --- | --- | --- |
| N | 2000 | 397 | 702 | 901 |  |
| **Demographic and anthropometric characteristics** | | | | | |
| Male sex, n (%) | 1062 (53.1) | 199 (50.1) | 394 (56.1) | 469 (52.1) | 0.112 |
| Age, years | 70.00 [61.00, 79.00] | 68.00 [56.00, 79.00] | 70.00 [63.00, 79.00] | 71.00 [63.00, 79.00] | <0.001 |
| BMI, kg/m² | 24.22 [21.43, 26.59] | 24.22 [21.19, 26.57] | 23.91 [21.09, 26.41] | 24.34 [21.80, 26.85] | 0.024 |
| **Vital signs at admission** | | | | | |
| SBP, mmHg | 129.00 [114.00, 144.00] | 128.00 [115.00, 143.00] | 124.00 [108.00, 139.00] | 134.00 [119.00, 149.00] | <0.001 |
| DBP, mmHg | 75.00 [66.00, 87.00] | 75.00 [67.00, 87.00] | 74.00 [65.00, 85.00] | 77.00 [66.00, 87.00] | 0.031 |
| HR, bpm | 79.00 [69.00, 94.00] | 78.00 [67.00, 91.00] | 78.00 [68.00, 96.00] | 80.00 [70.00, 95.00] | 0.001 |
| **Laboratory measurements** | | | | | |
| BNP, pg/mL | 574.65 [214.08, 1159.25] | 499.00 [199.16, 962.73] | 548.00 [221.50, 1147.50] | 645.00 [237.00, 1320.00] | 0.001 |
| WBC, ×10^9/L | 6.20 [5.00, 7.80] | 5.90 [4.70, 7.10] | 6.05 [4.93, 7.60] | 6.50 [5.30, 8.20] | <0.001 |
| RDW, % | 13.60 [13.00, 14.60] | 13.40 [13.00, 14.60] | 13.70 [12.90, 14.80] | 13.60 [13.00, 14.50] | 0.207 |
| Hb, g/L | 128.00 [112.00, 142.00] | 129.00 [114.00, 143.00] | 131.00 [116.00, 143.00] | 126.00 [108.00, 139.00] | <0.001 |
| ALB, g/L | 38.20 [35.70, 40.60] | 38.80 [35.90, 41.20] | 38.40 [36.20, 40.80] | 37.60 [35.10, 40.10] | <0.001 |
| PLT, ×10^9/L | 172.00 [136.00, 220.00] | 174.00 [133.00, 219.00] | 168.00 [133.00, 214.00] | 176.00 [141.00, 225.00] | 0.033 |
| TC, mg/dL | 141.92 [116.01, 172.47] | 151.20 [124.13, 185.23] | 144.24 [116.82, 173.18] | 136.89 [112.14, 168.61] | <0.001 |
| TG, mg/dL | 89.43 [68.18, 124.84] | 89.52 [69.70, 126.97] | 88.54 [67.29, 119.54] | 89.43 [68.18, 129.27] | 0.275 |
| HDL-C, mg/dL | 38.67 [31.71, 47.56] | 39.44 [33.26, 48.72] | 39.44 [32.10, 49.11] | 37.90 [30.94, 46.02] | <0.001 |
| LDL-C, mg/dL | 78.11 [58.01, 104.41] | 83.14 [66.13, 110.21] | 81.98 [57.62, 105.18] | 74.63 [53.75, 99.38] | <0.001 |
| ALT, U/L | 19.80 [13.10, 33.42] | 19.40 [13.10, 37.30] | 20.70 [13.50, 31.98] | 18.90 [12.80, 32.90] | 0.219 |
| AST, U/L | 23.70 [17.50, 34.12] | 24.10 [18.80, 36.48] | 24.41 [18.30, 33.85] | 22.40 [16.80, 33.00] | 0.003 |
| Cr, μmol/L | 83.35 [65.57, 112.85] | 77.00 [63.00, 100.90] | 82.50 [64.25, 104.52] | 88.00 [67.10, 126.00] | <0.001 |
| eGFR, mL/min/1.73 m² | 72.00 [49.00, 91.00] | 77.00 [58.00, 94.00] | 75.00 [54.00, 92.00] | 66.00 [42.00, 89.00] | <0.001 |
| K^+^, mmol/L | 3.90 [3.55, 4.22] | 3.89 [3.59, 4.16] | 3.91 [3.60, 4.21] | 3.90 [3.52, 4.25] | 0.577 |
| Na^+^, mmol/L | 139.00 [136.20, 141.70] | 139.20 [136.80, 141.60] | 139.00 [136.60, 141.78] | 139.00 [135.80, 141.70] | 0.623 |
| UA, μmol/L | 428.00 [338.65, 533.62] | 411.00 [331.00, 519.00] | 435.50 [339.00, 529.45] | 428.20 [341.00, 538.00] | 0.255 |
| LDH, U/L | 237.35 [198.00, 302.00] | 246.00 [199.50, 299.00] | 238.00 [203.00, 296.67] | 234.00 [192.90, 307.00] | 0.292 |
| FPG, mg/dL | 90.75 [81.36, 111.47] | 82.69 [77.50, 88.92] | 88.56 [80.61, 100.08] | 111.96 [90.00, 144.06] | <0.001 |
| Total protein, g/L | 63.30 [58.98, 68.10] | 63.80 [59.40, 69.10] | 63.15 [59.20, 67.70] | 63.10 [58.30, 67.90] | 0.190 |
| CRP, mg/L | 4.50 [3.00, 10.93] | 4.30 [2.90, 11.63] | 4.50 [3.10, 10.60] | 4.60 [2.92, 11.00] | 0.825 |
| TyG index | 8.39 [8.04, 8.81] | 8.26 [7.92, 8.58] | 8.30 [7.97, 8.61] | 8.60 [8.14, 9.10] | <0.001 |
| CTI index | 9.12 [8.63, 9.69] | 8.86 [8.45, 9.34] | 9.02 [8.60, 9.50] | 9.43 [8.85, 9.99] | <0.001 |
| HbA1c, % | 6.10 [5.63, 6.80] | 5.40 [5.20, 5.56] | 5.96 [5.80, 6.10] | 6.90 [6.50, 8.00] | <0.001 |
| **Echocardiographic parameters** | | | | | |
| LVDd, cm | 5.80 [5.15, 6.50] | 5.85 [5.16, 6.70] | 5.85 [5.10, 6.60] | 5.75 [5.17, 6.47] | 0.158 |
| LAD, cm | 4.60 [4.00, 5.16] | 4.64 [3.99, 5.15] | 4.70 [4.10, 5.25] | 4.57 [4.00, 5.10] | 0.035 |
| LVEF, % | 43.00 [34.00, 52.00] | 42.00 [31.90, 51.30] | 43.00 [33.05, 52.40] | 43.00 [35.00, 53.00] | 0.107 |
| **Comorbidities and medical history** | | | | | |
| Coronary artery disease, n (%) | 468 (23.4) | 102 (25.7) | 193 (27.5) | 173 (19.2) | <0.001 |
| Atrial fibrillation, n (%) | 880 (44.0) | 166 (41.8) | 348 (49.6) | 366 (40.6) | 0.001 |
| Hyperlipidaemia, n (%) | 367 (18.4) | 69 (17.4) | 96 (13.7) | 202 (22.4) | <0.001 |
| Hypertension, n (%) | 1225 (61.3) | 204 (51.4) | 366 (52.1) | 655 (72.7) | <0.001 |
| COPD, n (%) | 87 (4.3) | 21 (5.3) | 30 (4.3) | 36 (4.0) | 0.548 |
| **Baseline medications** | | | | | |
| Statin, n (%) | 1112 (55.6) | 188 (47.4) | 369 (52.6) | 555 (61.6) | <0.001 |
| Antiplatelet agent, n (%) | 1040 (52.0) | 204 (51.4) | 335 (47.7) | 501 (55.6) | 0.007 |
| MRA, n (%) | 1091 (54.5) | 216 (54.4) | 409 (58.3) | 466 (51.7) | 0.033 |
| Beta-blocker, n (%) | 1533 (76.6) | 301 (75.8) | 538 (76.6) | 694 (77.0) | 0.887 |
| Diuretic, n (%) | 1723 (86.2) | 322 (81.1) | 619 (88.2) | 782 (86.8) | 0.005 |
| ACEI/ARB/ARNI, n (%) | 944 (47.2) | 178 (44.8) | 341 (48.6) | 425 (47.2) | 0.492 |
| Digoxin, n (%) | 273 (13.7) | 67 (16.9) | 116 (16.5) | 90 (10.0) | <0.001 |
| SGLT2 inhibitor, n (%) | 342 (17.1) | 38 (9.6) | 84 (12.0) | 220 (24.4) | <0.001 |
| Abbreviations: SBP, systolic blood pressure; DBP, diastolic blood pressure; HR, heart rate; BMI, body mass index; BNP, B-type natriuretic peptide; WBC, white blood cell; RDW, red cell distribution width; Hb, haemoglobin; ALB, albumin; PLT, platelet; TC, total cholesterol; TG, triglycerides; ALT, alanine aminotransferase; AST, aspartate aminotransferase; Cr, creatinine; eGFR, estimated glomerular filtration rate; K^+^, potassium; Na^+^, sodium; UA, uric acid; LDH, lactate dehydrogenase; FPG, fasting plasma glucose; CRP, C-reactive protein; TyG, triglyceride–glucose index; CTI, C-reactive protein–triglyceride–glucose index; HDL-C, high-density lipoprotein cholesterol; LDL-C, low-density lipoprotein cholesterol; HbA1c, glycated haemoglobin A1c; LVDd, left ventricular end-diastolic diameter; LAD, left atrial diameter; LVEF, left ventricular ejection fraction; CAD, coronary artery disease; AF, atrial fibrillation; COPD, chronic obstructive pulmonary disease; MRA, mineralocorticoid receptor antagonist; ACEI, angiotensin-converting enzyme inhibitor; ARB, angiotensin receptor blocker; ARNI, angiotensin receptor-neprilysin inhibitor; SGLT2i, sodium–glucose cotransporter 2 inhibitor. | | | | | |

**Table S2. Associations of continuous CTI per 1-SD increase with all-cause mortality and MACCEs in the DM and non-DM subgroups.**

| **Endpoint** | **Subgroup** | **Model** | **HR (95% CI)** | **P value** |
| --- | --- | --- | --- | --- |
| **All-cause mortality** | non-DM | Model 1 | 1.23 (1.06–1.43) | 0.006 |
|  |  | Model 2 | 1.33 (1.14–1.55) | <0.001 |
|  |  | Model 3 | 1.29 (1.10–1.52) | 0.002 |
|  | DM | Model 1 | 1.49 (1.30–1.71) | <0.001 |
|  |  | Model 2 | 1.51 (1.31–1.73) | <0.001 |
|  |  | Model 3 | 1.62 (1.40–1.87) | <0.001 |
| **MACCEs** | non-DM | Model 1 | 1.30 (1.15–1.46) | <0.001 |
|  |  | Model 2 | 1.35 (1.20–1.53) | <0.001 |
|  |  | Model 3 | 1.30 (1.15–1.48) | <0.001 |
|  | DM | Model 1 | 1.30 (1.16–1.45) | <0.001 |
|  |  | Model 2 | 1.32 (1.18–1.48) | <0.001 |
|  |  | Model 3 | 1.32 (1.18–1.48) | <0.001 |
| Abbreviations: CTI, C-reactive protein-triglyceride-glucose index; DM, diabetes mellitus; MACCEs, major adverse cardiac and cerebrovascular events; HR, hazard ratio; CI, confidence interval. Model 1: Unadjusted. Model 2: Adjusted for age and sex. Model 3: Adjusted for age, sex, BMI, SBP, HR, eGFR, LVEF, BNP, atrial fibrillation, coronary artery disease, and the use of SGLT2 inhibitors, ACEIs/ARBs/ARNIs, beta-blockers, MRAs, diuretics, and statins. | | | | |

**Table S3. Interaction between continuous CTI and diabetes status for all-cause mortality and MACCEs.**

| **Endpoint** | **Model** | **Interaction HR (95% CI)** | **P for interaction** |
| --- | --- | --- | --- |
| All-cause mortality | Model 1 | 1.21 (0.98–1.48) | 0.071 |
|  | Model 2 | 1.12 (0.91–1.38) | 0.272 |
|  | Model 3 | 1.18 (0.96–1.45) | 0.107 |
| MACCEs | Model 1 | 0.99 (0.84–1.17) | 0.901 |
|  | Model 2 | 0.96 (0.82–1.13) | 0.644 |
|  | Model 3 | 1.00 (0.85–1.18) | 0.997 |
| Abbreviations: CTI, C-reactive protein-triglyceride-glucose index; MACCEs, major adverse cardiac and cerebrovascular events; HR, hazard ratio; CI, confidence interval. Model 1: Unadjusted.  Model 2: Adjusted for age and sex.  Model 3: Adjusted for age, sex, BMI, SBP, HR, eGFR, LVEF, BNP, atrial fibrillation, coronary artery disease, and the use of SGLT2 inhibitors, ACEIs/ARBs/ARNIs, beta-blockers, MRAs, diuretics, and statins. | | | |

**Table S4. Multiplicative interaction between diabetes status and high versus low CTI level for all-cause mortality and MACCEs.**

| **Endpoint** | **Interaction HR (95% CI)** | **P for interaction** |
| --- | --- | --- |
| All-cause mortality | 1.51 (0.97–2.35) | 0.068 |
| MACCEs | 1.28 (0.90–1.83) | 0.165 |
| Abbreviations: CTI, C-reactive protein-triglyceride-glucose index; MACCEs, major adverse cardiac and cerebrovascular events; HR, hazard ratio; CI, confidence interval. The interaction was evaluated in the fully adjusted model. | | |

**Table S5. Associations of CTI with all-cause mortality and MACCEs according to glycaemic status.**

|  |  |  | **Model 1** | | | **Model 2** | | | **Model 3** | | |
| --- | --- | --- | --- | --- | --- | --- | --- | --- | --- | --- | --- |
| **Glycaemic status** | **No. of patients** | **No. of events** | **HR (95% CI)** | **P value** | **P for interaction** | **HR (95% CI)** | **P value** | **P for interaction** | **HR (95% CI)** | **P value** | **P for interaction** |
| **All-cause mortality** |  |  |  |  |  |  |  |  |  |  |  |
| **CTI as continuous variable (per 1-SD)** |  |  |  |  | **0.119** |  |  | **0.085** |  |  | **0.110** |
| Normoglycaemia | 397 | 60 | 1.06 (0.76–1.47) | 0.736 |  | 1.08 (0.76–1.53) | 0.684 |  | 1.02 (0.71–1.48) | 0.908 |  |
| Prediabetes | 702 | 127 | 1.43 (1.15–1.76) | 0.001 |  | 1.33 (1.07–1.65) | 0.010 |  | 1.36 (1.09–1.69) | 0.007 |  |
| Diabetes | 901 | 179 | 1.48 (1.30–1.69) | <0.001 |  | 1.48 (1.30–1.69) | <0.001 |  | 1.57 (1.37–1.79) | <0.001 |  |
| **CTI as categorical variable (High vs. Low)** |  |  |  |  | **0.657** |  |  | **0.387** |  |  | **0.388** |
| Normoglycaemia | 397 | 60 | 1.37 (0.74–2.53) | 0.322 |  | 1.39 (0.72–2.67) | 0.323 |  | 1.21 (0.61–2.39) | 0.592 |  |
| Prediabetes | 702 | 127 | 1.84 (1.27–2.67) | 0.001 |  | 1.63 (1.11–2.39) | 0.012 |  | 1.62 (1.09–2.38) | 0.016 |  |
| Diabetes | 901 | 179 | 1.77 (1.31–2.39) | <0.001 |  | 1.85 (1.37–2.51) | <0.001 |  | 1.98 (1.45–2.70) | <0.001 |  |
| **MACCEs** |  |  |  |  |  |  |  |  |  |  |  |
| **CTI as continuous variable (per 1-SD)** |  |  |  |  | **0.844** |  |  | **0.948** |  |  | **0.945** |
| Normoglycaemia | 397 | 77 | 1.38 (1.06–1.80) | 0.017 |  | 1.51 (1.13–2.03) | 0.006 |  | 1.45 (1.06–1.97) | 0.018 |  |
| Prediabetes | 702 | 193 | 1.36 (1.15–1.61) | <0.001 |  | 1.33 (1.12–1.57) | 0.001 |  | 1.32 (1.11–1.57) | 0.002 |  |
| Diabetes | 901 | 281 | 1.31 (1.18–1.45) | <0.001 |  | 1.28 (1.15–1.42) | <0.001 |  | 1.31 (1.18–1.46) | <0.001 |  |
| **CTI as categorical variable (High vs. Low)** |  |  |  |  | **0.693** |  |  | **0.530** |  |  | **0.532** |
| Normoglycaemia | 397 | 77 | 1.23 (0.71–2.14) | 0.466 |  | 1.36 (0.76–2.45) | 0.302 |  | 1.20 (0.66–2.19) | 0.556 |  |
| Prediabetes | 702 | 193 | 1.63 (1.21–2.21) | 0.001 |  | 1.53 (1.13–2.09) | 0.007 |  | 1.50 (1.10–2.06) | 0.011 |  |
| Diabetes | 901 | 281 | 1.60 (1.26–2.03) | <0.001 |  | 1.53 (1.20–1.95) | <0.001 |  | 1.61 (1.26–2.06) | <0.001 |  |
| Abbreviations: HR, hazard ratio; CI, confidence interval; SD, standard deviation; CTI, C-reactive protein-triglyceride-glucose index; DM, diabetes mellitus; MACCEs, major adverse cardiac and cerebrovascular events; Model 1: Unadjusted.  Model 2: Adjusted for age and sex.  Model 3: Adjusted for age, sex, BMI, SBP, HR, eGFR, LVEF, BNP, atrial fibrillation, coronary artery disease, and the use of SGLT2 inhibitors, ACEIs/ARBs/ARNIs, beta-blockers, MRAs, diuretics, and statins. | | | | | | | | | | | |

**Table S6. Associations of diabetes status and CTI level with all-cause mortality stratified by SGLT2 inhibitor use.**

| **Variable** | **Non-SGLT2i group (n = 1658)** |  | **SGLT2i group (n = 342)** |  |
| --- | --- | --- | --- | --- |
|  | **HR (95% CI)** | **P value** | **HR (95% CI)** | **P value** |
| Group |  |  |  |  |
| non-DM + low CTI | 1.00 (reference) | – | 1.00 (reference) | – |
| non-DM + high CTI | 1.40 (1.01–1.93) | 0.043 | 2.12 (1.10–4.21) | 0.039 |
| DM + low CTI | 0.87 (0.61–1.22) | 0.412 | 0.87 (0.45–1.70) | 0.688 |
| DM + high CTI | 2.23 (1.63–3.04) | <0.001 | 1.05 (0.54–2.05) | 0.886 |
| P for interaction |  | 0.048 |  |  |
| Abbreviations: HR, hazard ratio; CI, confidence interval; DM, diabetes mellitus; CTI, C-reactive protein-triglyceride-glucose index; SGLT2i, sodium-glucose cotransporter-2 inhibitors;  Models were adjusted for Model 3 covariates except SGLT2 inhibitor use.  The nominal P for interaction by SGLT2 inhibitor use was 0.048; the corresponding FDR-adjusted P was 0.651 (Table S12). | | | | |

**Table S7. Fine–Gray competing-risk regression for MACCEs with all-cause mortality as a competing event.**

| **Variable** | **SHR (95% CI)** | **P value** |
| --- | --- | --- |
| Group | | |
| non-DM + low CTI | 1.00 (reference) | – |
| non-DM + high CTI | 1.38 (1.09–1.76) | 0.009 |
| DM + low CTI | 1.10 (0.87–1.39) | 0.420 |
| DM + high CTI | 1.67 (1.33–2.10) | <0.001 |
| Abbreviations: MACCEs, major adverse cardiac and cerebrovascular events; SHR, subdistribution hazard ratio; CI, confidence interval; DM, diabetes mellitus; CTI, C-reactive protein-triglyceride-glucose index;  The model was adjusted for the covariates included in Model 3. | | |

Table S8. Thirty-day landmark analysis for all-cause mortality and MACCEs according to diabetes status and CTI level.

| **Variable** | **HR (95% CI)** | **P value** |
| --- | --- | --- |
| **All-cause mortality** |  |  |
| non-DM + low CTI | 1.00 (reference) | – |
| non-DM + high CTI | 1.58 (1.16–2.15) | 0.004 |
| DM + low CTI | 0.95 (0.70–1.31) | 0.767 |
| DM + high CTI | 1.73 (1.28–2.32) | <0.001 |
| **MACCEs** |  |  |
| non-DM + low CTI | 1.00 (reference) | – |
| non-DM + high CTI | 1.41 (1.08–1.83) | 0.010 |
| DM + low CTI | 1.22 (0.96–1.56) | 0.110 |
| DM + high CTI | 1.67 (1.31–2.14) | <0.001 |
| Abbreviations: HR, hazard ratio; CI, confidence interval; DM, diabetes mellitus; CTI, C-reactive protein-triglyceride-glucose index; MACCEs, major adverse cardiac and cerebrovascular events.  Models were adjusted for the covariates included in Model 3. | | |

Table S9. Sensitivity analysis using stratified Cox models to account for potential violations of the proportional hazards assumption.

|  | **All-cause mortality** | | **MACCEs** | |
| --- | --- | --- | --- | --- |
| **Variable** | **HR (95% CI)** | **P value** | **HR (95% CI)** | **P value** |
| **Group** |  |  |  |  |
| non-DM + low CTI | 1.00 (reference) | – | 1.00 (reference) | – |
| non-DM + high CTI | 1.66 (1.21–2.27) | 0.002 | 1.45 (1.12–1.88) | 0.005 |
| DM + low CTI | 0.81 (0.59–1.13) | 0.216 | 1.13 (0.89–1.45) | 0.320 |
| DM + high CTI | 1.97 (1.45–2.68) | <0.001 | 2.05 (1.60–2.63) | <0.001 |
| Abbreviations: HR, hazard ratio; CI, confidence interval; MACCEs, major adverse cardiac and cerebrovascular events; DM, diabetes mellitus; CTI, C-reactive protein-triglyceride-glucose index;  Stratified Cox models were adjusted for the covariates included in Model 3, with stratification applied to variables violating the proportional hazards assumption. | | | | |

**Table S10. Sensitivity analyses excluding patients with elevated inflammatory markers.**

| **Sensitivity analysis** | **Outcome** | **Group** | **Events/Total** | **Adjusted HR (95% CI)** | **P value** |
| --- | --- | --- | --- | --- | --- |
| Excluding CRP >20 mg/L | All-cause mortality | non-DM + low CTI | 111/769 | 1.00 (reference) | — |
|  |  | non-DM + high CTI | 59/268 | 1.62 (1.17–2.25) | 0.004 |
|  |  | DM + low CTI | 49/340 | 0.90 (0.63–1.27) | 0.536 |
|  |  | DM + high CTI | 81/292 | 2.14 (1.58–2.92) | <0.001 |
|  | MACCEs | non-DM + low CTI | 177/769 | 1.00 (reference) | — |
|  |  | non-DM + high CTI | 90/268 | 1.50 (1.16–1.95) | 0.002 |
|  |  | DM + low CTI | 88/340 | 1.06 (0.82–1.38) | 0.648 |
|  |  | DM + high CTI | 105/292 | 1.74 (1.34–2.25) | <0.001 |
| Excluding CRP >50 mg/L | All-cause mortality | non-DM + low CTI | 123/836 | 1.00 (reference) | — |
|  |  | non-DM + high CTI | 67/306 | 1.61 (1.19–2.18) | 0.002 |
|  |  | DM + low CTI | 57/383 | 0.90 (0.65–1.25) | 0.540 |
|  |  | DM + high CTI | 89/321 | 2.11 (1.58–2.83) | <0.001 |
|  | MACCEs | non-DM + low CTI | 192/836 | 1.00 (reference) | — |
|  |  | non-DM + high CTI | 96/306 | 1.44 (1.12–1.84) | 0.005 |
|  |  | DM + low CTI | 102/383 | 1.11 (0.86–1.42) | 0.420 |
|  |  | DM + high CTI | 124/321 | 1.96 (1.54–2.49) | <0.001 |
| Excluding WBC >10 ×10⁹/L | All-cause mortality | non-DM + low CTI | 133/859 | 1.00 (reference) | — |
|  |  | non-DM + high CTI | 59/277 | 1.48 (1.09–2.02) | 0.013 |
|  |  | DM + low CTI | 61/398 | 0.85 (0.62–1.17) | 0.327 |
|  |  | DM + high CTI | 65/282 | 1.61 (1.17–2.22) | 0.003 |
|  | MACCEs | non-DM + low CTI | 191/859 | 1.00 (reference) | — |
|  |  | non-DM + high CTI | 79/277 | 1.34 (1.03–1.75) | 0.031 |
|  |  | DM + low CTI | 106/398 | 1.14 (0.90–1.46) | 0.282 |
|  |  | DM + high CTI | 98/282 | 1.90 (1.47–2.47) | <0.001 |
| Abbreviations: HR, hazard ratio; CI, confidence interval; MACCEs, major adverse cardiac and cerebrovascular events; DM, diabetes mellitus; CTI, C-reactive protein-triglyceride-glucose index; CRP, C-reactive protein; WBC, white blood cell count.  Models were adjusted for age, sex, BMI, systolic blood pressure, heart rate, eGFR, LVEF, BNP, atrial fibrillation, coronary artery disease, and baseline cardiovascular medications, including SGLT2 inhibitor, ACEI/ARB/ARNI, beta-blocker, MRA, diuretic, and statin use. | | | | | |

**Table S11. Distribution of qualifying CKM-related abnormalities according to diabetes status and CTI level.**

| **Variable** | **Overall** | **non-DM + low CTI** | **non-DM + high CTI** | **DM + low CTI** | **DM + high CTI** | **P value** |
| --- | --- | --- | --- | --- | --- | --- |
| N | 2000 | 907 | 332 | 417 | 344 | — |
| Dysglycaemia | 1603 (80.2) | 586 (64.6) | 256 (77.1) | 417 (100.0) | 344 (100.0) | <0.001 |
| Diabetes mellitus | 761 (38.0) | 0 (0.0) | 0 (0.0) | 417 (100.0) | 344 (100.0) | — |
| Hypertension | 1390 (69.5) | 568 (62.6) | 193 (58.1) | 344 (82.5) | 285 (82.8) | <0.001 |
| Hyperlipidaemia | 1675 (83.8) | 692 (76.3) | 305 (91.9) | 356 (85.4) | 322 (93.6) | <0.001 |
| Chronic kidney disease | 722 (36.1) | 261 (28.8) | 117 (35.2) | 165 (39.6) | 179 (52.0) | <0.001 |
| Adiposity abnormality | 814 (40.7) | 355 (39.1) | 140 (42.2) | 169 (40.5) | 150 (43.6) | 0.490 |
| Number of qualifying CKM-related abnormalities | 3 (2, 4) | 3 (2, 3) | 3 (2, 4) | 3 (3, 4) | 4 (3, 4) | <0.001 |
| CKM burden category |  |  |  |  |  | <0.001 |
| 1 | 151 (7.5) | 125 (13.8) | 21 (6.3) | 3 (0.7) | 2 (0.6) |  |
| 2 | 397 (19.9) | 244 (26.9) | 86 (25.9) | 46 (11.0) | 21 (6.1) |  |
| ≥3 | 1452 (72.6) | 538 (59.3) | 225 (67.8) | 368 (88.2) | 321 (93.3) |  |
| Values are presented as n (%) or median (interquartile range). CKM burden was calculated as the number of qualifying non-cardiovascular CKM abnormalities: dysglycaemia, hypertension, hyperlipidaemia, chronic kidney disease, and adiposity abnormality. In this table, hypertension and hyperlipidaemia refer to qualifying CKM-related abnormalities rather than documented history alone. Hypertension was defined as SBP ≥140 mmHg, DBP ≥90 mmHg, current antihypertensive medication use, or documented prior diagnosis. Hyperlipidaemia was defined as TC ≥240 mg/dL, LDL-C ≥160 mg/dL, TG ≥150 mg/dL, HDL-C <40 mg/dL, or ongoing lipid-lowering therapy. Dysglycaemia was defined as FPG ≥100 mg/dL, HbA1c ≥5.7%, or documented diabetes history. Diabetes mellitus in this table refers to documented prior diagnosis and was used for the primary diabetes/CTI grouping. Adiposity abnormality was defined as BMI ≥25 kg/m². CKM burden was summarized as 1, 2, and ≥3 abnormalities, consistent with the cohort eligibility definition requiring at least one qualifying non-cardiovascular CKM abnormality. P values were not calculated for variables used to define the diabetes/CTI groups. CKM, cardiovascular–kidney–metabolic; CTI, C-reactive protein–triglyceride–glucose index; DM, diabetes mellitus; FPG, fasting plasma glucose; HbA1c, glycated haemoglobin A1c; IQR, interquartile range. | | | | | | |

**Table S12. Nominal and Benjamini–Hochberg false discovery rate-adjusted P values for exploratory subgroup interaction tests.**

| **Exposure contrast** | **Outcome** | **Subgroup** | **P for interaction** | **FDR-adjusted P** |
| --- | --- | --- | --- | --- |
| DM + high CTI | All-cause mortality | Age | 0.849 | 0.986 |
|  |  | Sex | 0.513 | 0.947 |
|  |  | BMI | 0.118 | 0.651 |
|  |  | Hypertension | 0.930 | 0.986 |
|  |  | SGLT2i use | 0.048 | 0.651 |
|  |  | LVEF category | 0.221 | 0.663 |
|  | MACCEs | Age | 0.700 | 0.986 |
|  |  | Sex | 0.339 | 0.814 |
|  |  | BMI | 0.888 | 0.986 |
|  |  | Hypertension | 0.277 | 0.739 |
|  |  | SGLT2i use | 0.495 | 0.947 |
|  |  | LVEF category | 0.958 | 0.986 |
| Non-DM + high CTI | All-cause mortality | Age | 0.984 | 0.986 |
|  |  | Sex | 0.937 | 0.986 |
|  |  | BMI | 0.092 | 0.651 |
|  |  | Hypertension | 0.085 | 0.651 |
|  |  | SGLT2i use | 0.183 | 0.651 |
|  |  | LVEF category | 0.190 | 0.651 |
|  | MACCEs | Age | 0.665 | 0.986 |
|  |  | Sex | 0.570 | 0.977 |
|  |  | BMI | 0.758 | 0.986 |
|  |  | Hypertension | 0.986 | 0.986 |
|  |  | SGLT2i use | 0.175 | 0.651 |
|  |  | LVEF category | 0.446 | 0.947 |

Nominal interaction P values were obtained from the adjusted Cox subgroup analyses presented in Figure 4 and Figure S4. To account for multiple testing, the 24 interaction P values, comprising six subgroup variables, two outcomes, and two exposure contrasts, were adjusted using the Benjamini-Hochberg false discovery rate procedure. None of the subgroup interactions remained statistically significant at an FDR-adjusted P value <0.05. BMI, body mass index; CTI, C-reactive protein-triglyceride-glucose index; DM, diabetes mellitus; FDR, false discovery rate; LVEF, left ventricular ejection fraction; MACCEs, major adverse cardiac and cerebrovascular events; SGLT2i, sodium-glucose cotransporter 2 inhibitor.

**Table S13. Complete-case sensitivity analyses of the associations of continuous CTI and diabetes/CTI categories with all-cause mortality and MACCEs.**

| **Outcome** | **Analysis** | **Group/Subgroup** | **Events/Total** | **Adjusted HR (95% CI)** | **P value** |
| --- | --- | --- | --- | --- | --- |
| All-cause mortality | Diabetes/CTI classification | non-DM + low CTI | 112/714 | 1.00 (reference) | — |
|  |  | non-DM + high CTI | 62/261 | 1.65 (1.20–2.27) | 0.002 |
|  |  | DM + low CTI | 49/348 | 0.78 (0.55–1.10) | 0.154 |
|  |  | DM + high CTI | 74/265 | 1.94 (1.42–2.66) | <0.001 |
|  | CTI per 1-SD increase | non-DM | 174/975 | 1.24 (1.04–1.48) | 0.018 |
|  |  | DM | 123/613 | 1.59 (1.36–1.86) | <0.001 |
| MACCEs | Diabetes/CTI classification | non-DM + low CTI | 168/714 | 1.00 (reference) | — |
|  |  | non-DM + high CTI | 76/261 | 1.31 (0.99–1.73) | 0.054 |
|  |  | DM + low CTI | 92/348 | 1.07 (0.82–1.39) | 0.630 |
|  |  | DM + high CTI | 97/265 | 1.85 (1.42–2.40) | <0.001 |
|  | CTI per 1-SD increase | non-DM | 244/975 | 1.28 (1.11–1.48) | <0.001 |
|  |  | DM | 189/613 | 1.32 (1.16–1.50) | <0.001 |

Values are adjusted hazard ratios (HRs) with 95% confidence intervals (CIs). Complete cases were participants with complete data for the exposure, outcomes, and all covariates included in the fully adjusted model. Models were adjusted for age, sex, BMI, systolic blood pressure, heart rate, eGFR, LVEF, BNP, atrial fibrillation, coronary artery disease, and baseline use of SGLT2 inhibitors, ACEIs/ARBs/ARNIs, beta-blockers, MRAs, diuretics, and statins. The 1-SD increment was based on the CTI standard deviation in the overall cohort (0.804). CTI, C-reactive protein–triglyceride–glucose index; DM, diabetes mellitus; MACCEs, major adverse cardiac and cerebrovascular events.
